# Homoisoflavanone Delays Colorectal Cancer Progression via DNA Damage‐Induced Mitochondrial Apoptosis and Parthanatos‐Like Cell Death

**DOI:** 10.1002/advs.202511406

**Published:** 2026-01-28

**Authors:** Hongjie Fan, Huzi Zhao, Pei Zhang, Pengfei Yu, Yunfei Ji, Gang Chen, Hongli Jin, Yanfang Liu, Jin Liu, Zhe‐Sheng Chen, Aiping Lyu, Xinmiao Liang, Yang Chen

**Affiliations:** ^1^ State Key Laboratory of Phytochemistry and Natural Medicines Dalian Institute of Chemical Physics Chinese Academy of Sciences Dalian China; ^2^ Ganjiang Chinese Medicine Innovation Center Nanchang China; ^3^ Department of Pathology Hubei Key Laboratory of Embryonic Stem Cell Research School of Basic Medical Science Hubei University of Medicine Shiyan China; ^4^ Statistics Program Department of Mathematics University of Maryland, College Park USA; ^5^ College of Food and Health Zhejiang Agriculture and Forest University Hangzhou China; ^6^ LawSau Fai Institute For Advancing Translational Medicine in Bone and Joint Diseases School of Chinese Medicine Hong Kong Baptist University Hong Kong SAR China; ^7^ Institute of Systems Medicine and Health Sciences School of Chinese Medicine Hong Kong Baptist University Hong Kong SAR China; ^8^ Department of Pharmaceutical Sciences College of Pharmacy and Health Sciences St. John's University Queens New York USA

**Keywords:** apoptosis, colorectal cancer, DNA damage, homoisoflavanone, parthanatos

## Abstract

Colorectal cancer remains a major global health challenge, particularly in advanced stages where current therapies show limited efficacy. Natural products, specifically those derived from herbal medicines, provide a valuable resource for discovering novel anticancer agents. In this study, a bioactive homoisoflavanone was successfully isolated and structurally characterized from *Polygonatum kingianum*, a widely used medicinal herb. In vitro, homoisoflavanone exhibited potent antiproliferative and pro‐apoptotic effects in colorectal cancer cells. Mechanistically, homoisoflavanon induced DNA damage mediated mitochondrial apoptosis and parthanatos‐like cell death, accompanied by ATM/ATR‐Chk1 pathway and PARP activation, loss of mitochondrial membrane potential, elevated ROS levels, and ATP depletion. In vivo, homoisoflavanone significantly suppressed tumor growth in a colorectal cancer xenograft model without inducing systemic toxicity. Immunohistochemical analysis further confirmed decreased proliferation, increased apoptosis, and parthanatos‐like cell death in tumor tissues. Collectively, these findings establish homoisoflavanone as a promising plant‐derived therapeutic candidate that targets DNA integrity and mitochondrial homeostasis to inhibit colorectal cancer progression, highlighting the potential of herbal medicine‐based compounds in anticancer drug development.

## Graphical abstract

Homoisoflavanone (HIF), a bioactive compound isolated from *Polygonatum kingianum*, selectively suppresses colorectal cancer progression by inducing DNA damage‐mediated mitochondrial apoptosis and parthanatos‐like cell death. HIF triggers mitochondrial dysfunction, including depolarized membrane potential, elevated ROS, and ATP depletion, while impairing DNA repair signaling. In vivo, HIF significantly inhibits tumor growth without systemic toxicity, highlighting its potential as a plant‐derived anticancer candidate.

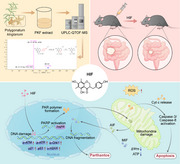

## 1 Introduction

Colorectal cancer (CRC) is among the most prevalent malignancies worldwide and remains a leading cause of cancer‐related mortality [1]. According to global cancer statistics, CRC accounts for nearly 10% of all newly diagnosed cancer cases and deaths each year, representing a major public health burden [2]. Although early‐stage CRC can often be managed successfully through surgical resection, patients with advanced or metastatic disease continue to face a poor prognosis due to drug resistance, recurrence, and limited therapeutic options [3, 4, 5]. Standard first‐line regimens such as FOLFOX (5‐fluorouracil (5‐FU), oxaliplatin, and leucovorin) and FOLFIRI (5‐FU, leucovorin, and irinotecan) in combination with bevacizumab have improved survival. However, their efficacy remains suboptimal and is frequently accompanied by cumulative toxicity, neuropathy, and myelosuppression [6]. Consequently, there is a critical need to discover new therapeutic agents that combine potent antitumor efficacy with improved safety profiles for long‐term management of CRC.

Natural products have long served as a cornerstone in the development of anticancer drugs [7]. Drug discovery based on these compounds has established valuable platforms for developing potent and low‐toxicity antitumor therapies. Historically, more than 60% of small‐molecule anticancer drugs approved by the U.S. Food and Drug Administration (FDA) are either natural products or derived from natural scaffolds [8]. These compounds often possess favorable pharmacological properties, including multitarget activity, structural diversity, and lower systemic toxicity compared to synthetic agents [9, 10]. Among them, herbal medicines constitute an especially rich resource for drug discovery because of their well‐documented clinical use, structural complexity, and ability to modulate multiple cellular pathways simultaneously [11]. The major bioactive classes of phytochemicals, such as flavonoids, alkaloids, terpenoids, and polysaccharides, have demonstrated broad antitumor potential across various cancer types, including CRC [12, 13, 14].

*Polygonatum kingianum* Coll. et Hemsl, belonging to the *Liliaceae* family, is a traditional Chinese medicinal herb widely used for its health‐promoting effects [15]. Classical Chinese pharmacopeias record its applications in strengthening the spleen, moistening the lungs, and nourishing the kidneys [16]. Modern pharmacological studies have revealed that *Polygonatum kingianum* exhibits diverse bioactivities, including anti‐inflammatory, antioxidant, immunomodulatory, and antitumor effects. Its therapeutic potential is attributed to a complex mixture of phytochemicals such as polysaccharides, saponins, alkaloids and flavonoids [17, 18]. Previous investigations have demonstrated that *Polygonatum kingianum* polysaccharides exert immunomodulatory and anti‐fatigue effects through gut microbiota modulation [19, 20], while dioscin, a saponin component, displays pronounced cytotoxicity against osteosarcoma cells with stem‐like properties [21]. However, despite increasing evidence of its pharmacological benefits, the chemical constituents and molecular mechanisms underlying the antitumor effects of *Polygonatum kingianum*‐derived flavonoids (PKF) remain poorly characterized.

Flavonoids represent one of the most extensively studied groups of plant secondary metabolites and are widely recognized for their antioxidant, anti‐inflammatory, and anticancer activities [12]. Numerous flavonoids have been identified to exert anti‐CRC activities through diverse mechanisms. For instance, baicalein, derived from *Scutellaria baicalensis*, suppresses colorectal tumorigenesis via inhibition of the TLR4 signaling pathway [22]. Kaempferol, found in various herbs, blocks colorectal cancer metastasis through regulation of the JMJD2C/β‐catenin signaling axis [23]. Additionally, luteolin, a polyphenolic flavonoid abundant in fruits, vegetables, and herbs, inhibits the proliferation of KRAS‐ and BRAF‐mutated colorectal cancer cells through apoptosis induction [24]. Despite these advances, the biological roles and molecular mechanisms of *Polygonatum kingianum*‐specific flavonoids, particularly homoisoflavanone, remain largely unexplored. Homoisoflavanone is a unique subclass of flavonoids characterized by a 3‐benzylchroman skeleton and exhibits diverse pharmacological properties, including antioxidant, anti‐inflammatory, and anticancer activities [25, 26, 27]. Given the long‐standing medicinal use of *Polygonatum kingianum* and the structural uniqueness of its homoisoflavanone constituents, investigating their antitumor potential holds significant promise for natural product‐based drug development.

In this study, we aimed to isolate and characterize a major bioactive component, 4',5,7‐trihydroxy‐6,8‐dimethylhomoisoflavanone (HIF), from the total flavonoid fraction of *Polygonatum kingianum* (PKF), and to evaluate their pharmacological activities against CRC both in vitro and in vivo. HIF was purified through phytochemical separation and structurally confirmed using liquid chromatography‐mass spectrometry (LC‐MS) and nuclear magnetic resonance (NMR) spectroscopy. Functional assays revealed that HIF exhibits potent antiproliferative, pro‐apoptotic, and anti‐migratory effects in CRC cell lines. Mechanistically, HIF induces cell cycle arrest, promotes DNA damage, and activates both mitochondrial apoptosis and parthanatos‐like cell death. Furthermore, HIF increases the expression of γ‐H2AX, phosphorylated ATM, ATR, and Chk1, indicating an effect on DNA damage response. In addition, HIF elevates mitochondrial reactive oxygen species (ROS), decreases membrane potential, and reduces ATP content, confirming profound mitochondrial dysfunction and oxidative stress‐mediated apoptosis. In vivo, both PKF and HIF significantly inhibited tumor growth in CRC xenograft mouse models without detectable systemic toxicity. Immunohistochemical analysis of tumor tissues confirmed reduced proliferation, enhanced apoptosis, and parthanatos‐like features. Together, these results identify HIF as a bioactive compound with potent antitumor activity against CRC.

In summary, this study isolates and characterizes a naturally occurring homoisoflavanone from *Polygonatum kingianum* and elucidates its role in inducing DNA damage, leading to mitochondrial apoptosis and parthanatos‐like cell death. These findings not only provide mechanistic insight into the pharmacological actions of *Polygonatum kingianum* but also highlight the potential of ethnopharmacology‐guided discovery of plant‐derived compounds as a valuable strategy for developing novel therapeutic agents against colorectal cancer.

## 2 Results

### 2.1 PKF Inhibits CRC Cell Growth by Inducing Apoptosis and Cell Cycle Arrest

**FIGURE 1:**
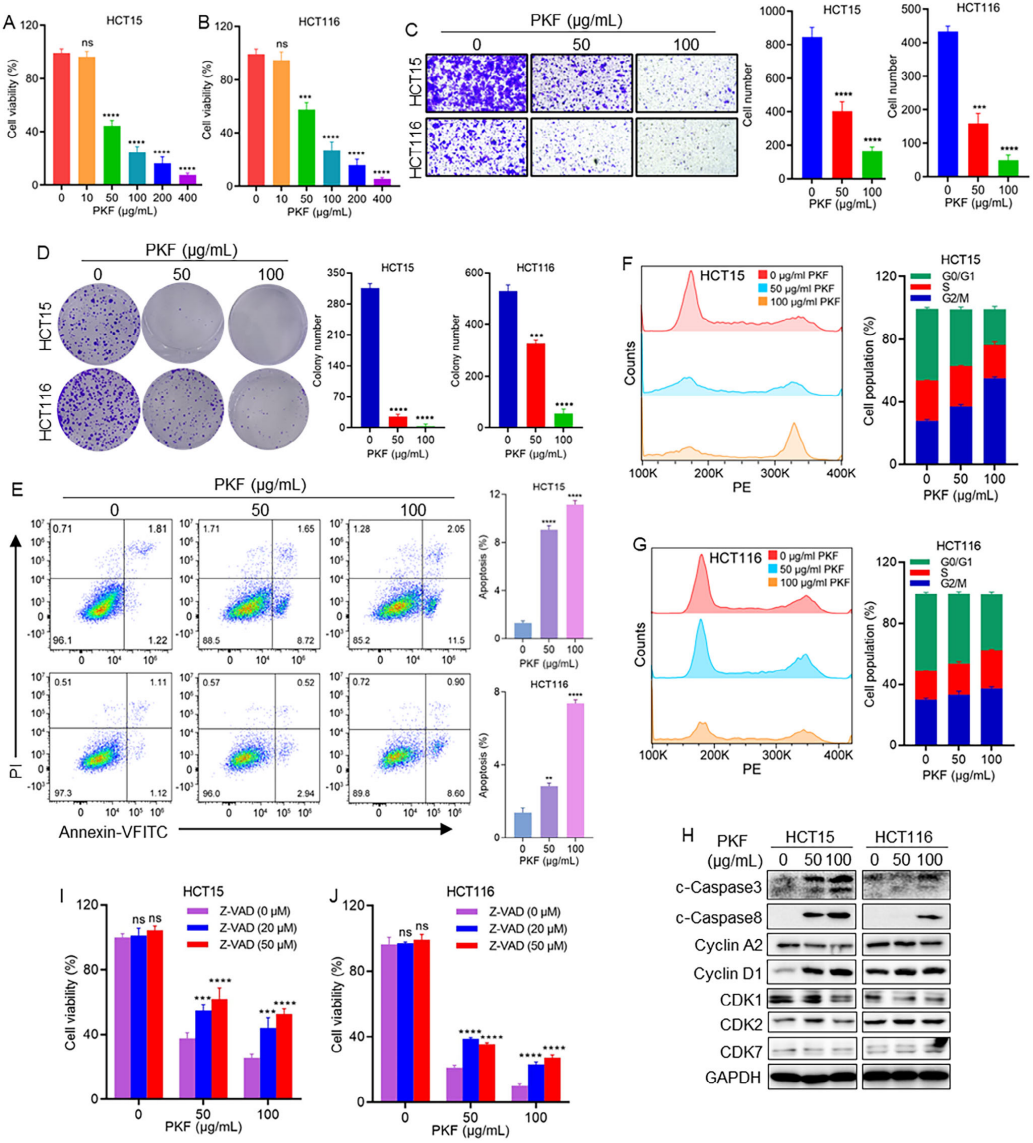
PKF inhibits CRC cell growth by inducing apoptosis and cell cycle arrest. (A, B) Cell viability of HCT15 and HCT116 cells treated with PKF for 24 h. (C) Cell migration of HCT15 and HCT116 cells after PKF treatment for 24. (D) Colony formation of HCT15 and HCT116 cells following PKF treatment for 24 h. (E) Cell apoptosis analysis of HCT15 and HCT116 cells after 24 h of PKF treatment. (F, G) Cell cycle analysis in HCT15 and HCT116 cells following PKF treatment for 24 h. (H) Protein expression levels of cell cycle‐related proteins (Cyclin D1, Cyclin A2, CDK1, CDK2, CDK7) and apoptosis markers (c‐Caspase‐3, c‐Caspase‐8) in HCT15 and HCT116 cells treated with PKF for 24 h. (I, J) Cell viability of HCT15 and HCT116 cells treated with PKF alone or in combination with Z‐VAD‐FMK (Z‐VAD). ^***^ *p* < 0.001, ^****^ *p* < 0.0001; ns, not significant.

To explore novel natural products with antitumor potential, total flavonoids were isolated from *Polygonatum kingianum* (PKF). Given the well‐documented anticancer properties of flavonoids, PKF was evaluated for its antiproliferative activity on CRC cells. Treatment of HCT15 and HCT116 cells with PKF treatment resulted in a significant dose‐dependent reduction of cell viability (Figure 1A,B). Furthermore, PKF markedly suppressed cell migration (Figure 1C) and inhibited clonogenic potential (Figure 1D) in both cell lines, indicating strong antiproliferative efficacy. Flow cytometry analysis revealed that PKF significantly induced apoptosis (Figure 1E) and caused cell cycle arrest at G2/M phase (Figure 1F,G). Western blot analysis further confirmed increased expression levels of cleaved Caspase‐3 (c‐Caspase‐3), cleaved Caspase‐8 (c‐Caspase‐8), Cyclin D1, and CDK2, alongside reduced expression of Cyclin A2 and CDK1, whereas CDK7 levels remained unchanged (Figure 1H). These results suggest that PKF interferes with cell cycle progression and activates apoptosis pathways in CRC cells. To further delineate the mechanisms of PKF‐induced cell death, we examined several canonical survival pathways. Notably, PKF treatment did not alter phosphorylated levels of AKT, ERK, and MEK (Figure S1A), indicating that its cytotoxic effects are independent of these signaling cascades. Functional rescue assays using pathway‐specific inhibitors demonstrated that Z‐VAD‐FMK (a pan‐caspase inhibitor) and Necrostatin‐1 (a necroptosis inhibitor) partially reversed PKF‐induced cell death, whereas Ferrostatin‐1 (a ferroptosis inhibitor) had no significant effect (Figure 1I,J; Figure S1B C). These findings demonstrate that PKF exerts potent antitumor activity against CRC cells primarily through apoptosis induction, cell cycle arrest, and partial necrotic cell death.

### 2.2 PKF Induces DNA Damage‐Mediated Apoptosis in CRC Cells

**FIGURE 2:**
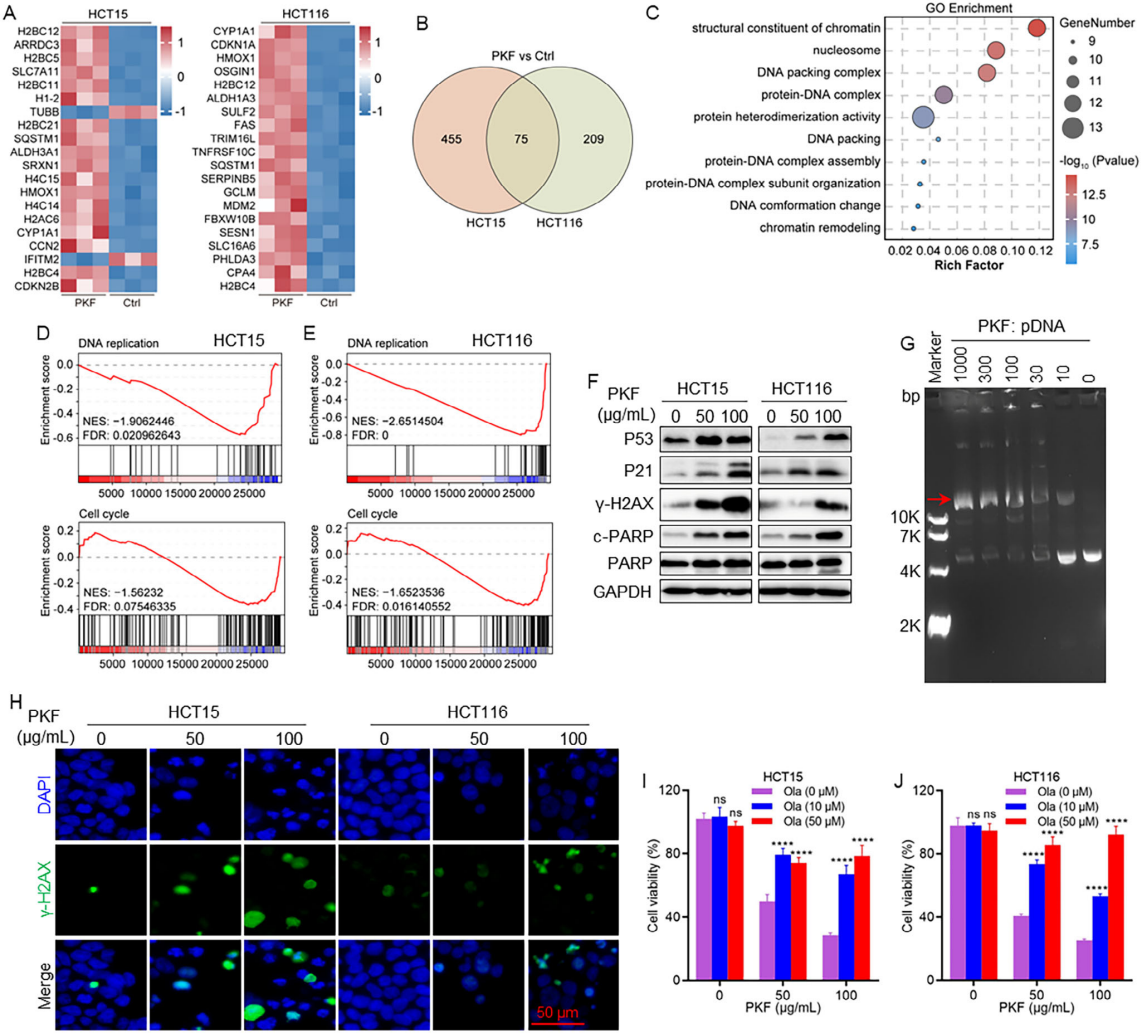
PKF induces DNA damage‐mediated apoptosis in CRC cells. (A) Heatmap showing differentially expressed genes in HCT15 and HCT116 cells treated with or without PKF. (B) Venn diagram displaying the overlap of differentially expressed genes between HCT15 and HCT116 cells. (C) Gene Ontology (GO) enrichment analysis of PKF‐regulated pathways in both cell lines. (D, E) Gene Set Enrichment Analysis (GSEA) of differentially expressed genes. (F) Protein expression levels of DNA damage markers (P53, P21, γ‐H2AX, c‐PARP, PARP) following PKF treatment. (G) Gel electrophoresis analysis of plasmid DNA incubated with PKF. The values represent the mass ratio of PKF to plasmid DNA, indicating that 200 ng of plasmid DNA was incubated with 0, 2, 6, 20, 60, and 200 µg of PKF for 30 min. (H) Immunofluorescence analysis of γ‐H2AX in CRC cells after PKF treatment. (I, J) Cell viability of HCT15 and HCT116 cells treated with PKF alone or in combination with Olaparib (Ola). ^****^ *p* < 0.0001; ns, not significant.

Transcriptome analysis was performed to elucidate the molecular mechanisms underlying the anticancer effects of PKF by comparing gene expression profiles between PKF‐treated and untreated CRC cells. Heatmap analysis revealed significant upregulation of cell cycle‐related genes, including *CDKN2B* and *CDKN1A*, as well as chromatin assembly‐related genes such as *H2BC12*, *H2BC4*, and *H2BC5* following PKF treatment (Figure 2A). Veen diagram analysis identified 75 differentially expressed genes commonly altered in both HCT15 and HCT116 cells (Figure 2B). Kyoto Encyclopedia of Genes and Genomes (KEGG) pathway enrichment analysis showed significant enrichment of the necrosis pathway (Figure S1D), suggesting its role in PKF‐induced cell death. Gene Ontology (GO) enrichment analysis further demonstrated significant enrichment in chromatin structural components and DNA packing complex (Figure 2C). Gene Set Enrichment Analysis (GSEA) indicated associations between PKF treatment and DNA replication and cell cycle regulation pathways (Figure 2D,E), suggesting that PKF may interfere with genomic stability. Consistently, immunoblotting analysis confirmed PKF‐induced upregulation of P53, P21, γ‐H2AX, and cleaved PARP (c‐PARP) in both cell lines (Figure 2F), validating activation of the DNA damage response pathway. DNA damage represents a fundamental cellular response to various stressors and can manifest in multiple forms, including base modifications, crosslinking, and single‐ or double‐strand breaks [28]. DNA gel electrophoresis analysis further revealed that PKF treatment converted supercoiled plasmid DNA into slower‐migrating nicked and linear forms, indicative of DNA strand breaks (Figure 2G). Immunofluorescence staining confirmed increased γ‐H2AX foci in both cell lines following PKF treatment (Figure 2H). To delineate the underlying cell death mechanisms, we performed rescue assays showing that Olaparib partially reversed PKF‐induced cytotoxicity, while its combination with Z‐VAD‐FMK achieved the greater rescue effect (Figure 2I,J; Figure S1E,F), indicating that PKF‐induced cytotoxicity involves a PARP‐dependent cell death pathway, consistent with features of DNA damage‐associated apoptosis.

### 2.3 PKF Treatment Effectively Suppresses CRC Tumor Growth in Vivo

**FIGURE 3:**
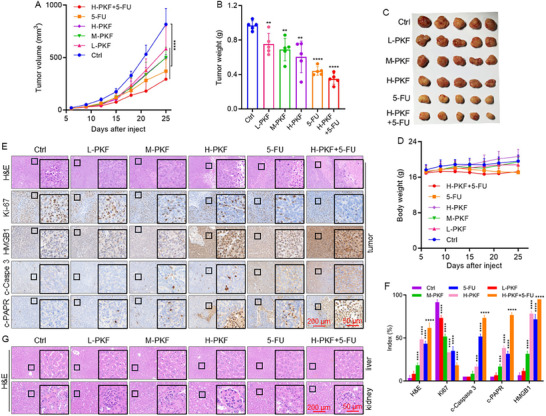
PKF treatment suppresses CRC tumor growth in vivo. (A) Tumor growth in mouse models subcutaneously injected with HCT116 cells and treated with PKF, 5‐FU, or a combination of PKF and 5‐FU. (B) Final tumor weights in each treatment group. (C) Representative images of tumors collected from each group. (D) Body weight monitoring of mice during treatment. (E, F) H&E and IHC staining of tumor tissues for Ki67, HMGB1, c‐Caspase‐3, and c‐PARP, with statistical analysis. (G) H&E and IHC staining of kidney and liver tissues to evaluate potential organ toxicity. ^**^ *p* < 0.01, ^***^ *p* < 0.001, ^****^ *p* < 0.0001.

Based on the potent anti‐CRC activity of PKF observed in vitro, we evaluated its therapeutic efficacy in vivo using a xenograft mouse model. HCT116 cells were subcutaneously injected into BALB/c nude mice. Seven days post‐injection, the mice were randomized into six groups: control, low‐dose PKF (L‐PKF), medium‐dose PKF (M‐PKF), high‐dose PKF (H‐PKF,) 5‐FU, and a combination of H‐PKF with 5‐FU. Treatments were administered daily via intraperitoneal injection. PKF significantly inhibited tumor growth, as evidenced by reductions in both tumor volume and weight compared to the control group. Moreover, PKF enhanced the antitumor effect of 5‐FU in the combination group (Figure 3A–C). Throughout the treatment period, body weights remained stable in PKF‐treated mice, suggesting no overt systemic toxicity (Figure 3D). Hematoxylin and eosin (H&E) staining of liver and kidney tissues revealed no pathological alterations, confirming the absence of major organ toxicity (Figure 3G). Immunohistochemical analysis of tumor tissues showed that PKF treatment markedly decreased the expression of the proliferation marker Ki67, while increasing the levels of high mobility group protein B1 (HMGB1), c‐PARP, and c‐Caspase‐3, suggesting that PKF suppresses CRC progression by inducing apoptosis and necrosis (Figure 3E,F).

### 2.4 HIF, the Key Compound From PKF, Inhibits the Growth of CRC Cells

**FIGURE 4:**
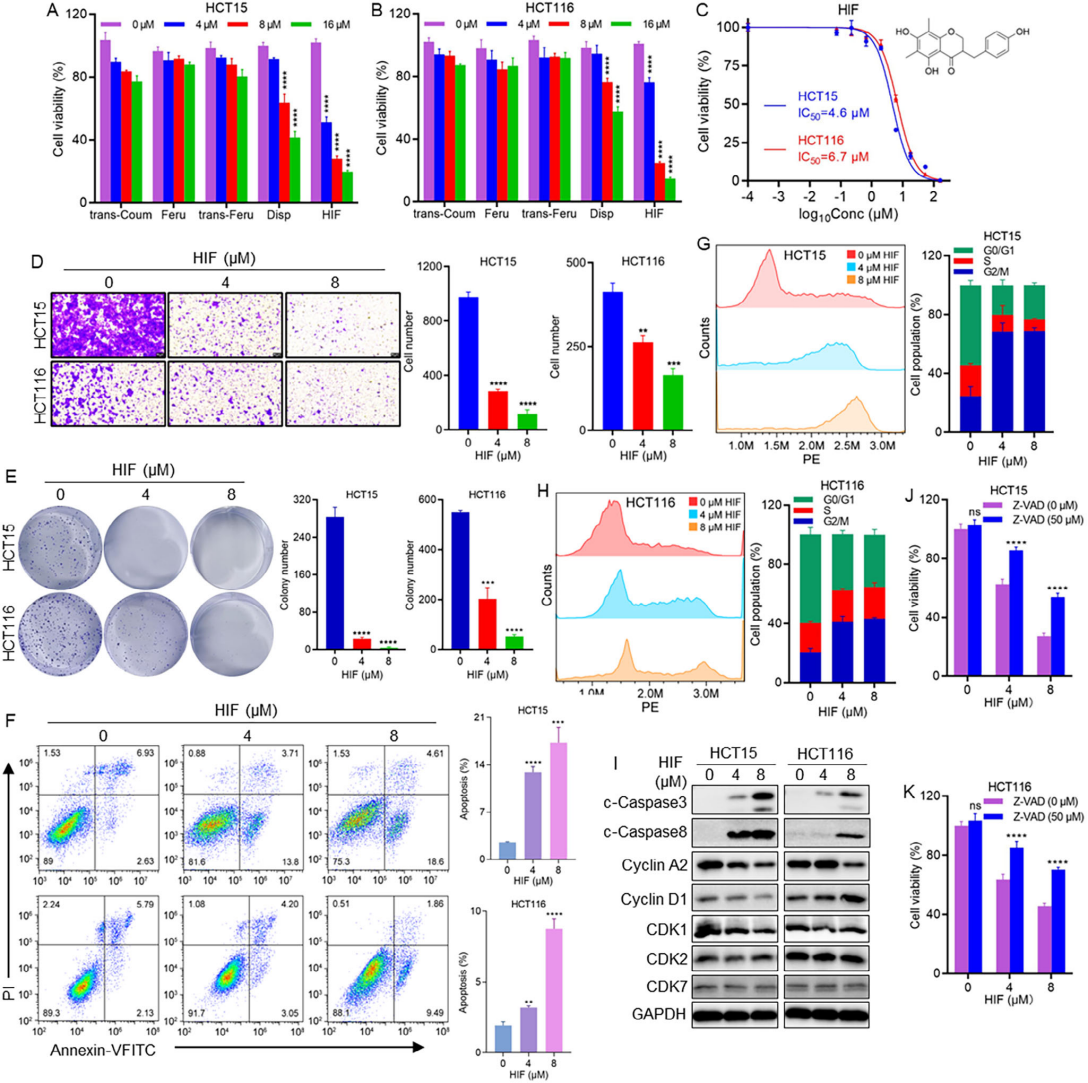
HIF inhibits CRC cells growth. (A, B) Cell viability of HCT15 and HCT116 cells treated with trans‐coumaroyltyramine (trans‐Coum), N‐feruloyloctopamine (Feru), N‐trans‐feruloyltyramine (trans‐Feru), disporopsin (Disp), or HIF for 24 h. (C) IC50 values of HIF in HCT15 and HCT116 cells. (D) Cell migration analysis of HCT15 and HCT116 cells following HIF treatment. (E) Colony formation assessment of HCT15 and HCT116 cells after HIF treatment for 24 h. (F) Apoptosis analysis of HCT15 and HCT116 cells after 24 h of HIF treatment. (G, H) Cell cycle distribution analysis of HCT15 and HCT116 cells following HIF treatment for 24 h. (I) Expression levels of cell cycle proteins (Cyclin D1, Cyclin A2, CDK1, CDK2, CDK7) and apoptosis markers (c‐Caspase3, c‐Caspase8). (J, K) The impact of Z‐VAD‐FMK (Z‐VAD) co‐treatment on HIF‐induced cell death in HCT15 and HCT116 cells. ^**^ *p* < 0.01, ^***^ *p* < 0.001, ^****^ *p* < 0.0001; ns, not significant.

To investigate the bioactive constituents of PKF, a botanical extract composed of multiple flavone compounds, we conducted ultra‐high‐performance liquid chromatography coupled with quadrupole time‐of‐flight mass spectrometry (UPLC‐QTOF‐MS). Five major compounds were identified: N‐FeruloyloctopaMine, N‐p‐trans‐Coumaroyltyramine, N‐trans‐Feruloyltyramine, disporopsin, and 4',5,7‐trihydroxy‐6,8‐dimethylhomoisoflavanone (HIF) (Figures S2 and S3). The anti‐CRC activity of these individual compounds was evaluated in HCT15 and HCT116 cells, among which HIF exhibited the most potent antiproliferative effect (Figure 4A,B). Structural elucidation of HIF was performed using NMR spectroscopy (Figures S4–S11). The half‐maximal inhibitory concentration (IC_50_) values of HIF were 4.6 µM for HCT15 cells and 6.7 µM for HCT116 cells (Figure 4C). Consistent with its antiproliferative activity, HIF significantly inhibited cell migration (Figure 4D) and reduced clonogenic potential in a dose‐dependent manner (Figure 4E). Flow cytometry analysis confirmed that HIF induced apoptosis (Figure 4F) and cell cycle arrest at G2/M phase (Figure 4G,H). Western blot analysis showed increased expression of c‐Caspase‐3, c‐Caspase‐8, Cyclin D1, accompanied by decreased levels of Cyclin A2, CDK1, and CDK2, while CDK7 expression remained unchanged following HIF treatment (Figure 4I), supporting its induction of apoptosis and cell cycle arrest.

To further elucidate the mechanisms underlying HIF‐induced cell death, cell death pathway inhibitors were employed in co‐treatment assays. Co‐treatment with Z‐VAD‐FMK partially rescued HIF‐induced cell death, while Necrostatin‐1 and Ferrostatin‐1 conferred no significant protective effects (Figure 4J,K; Figure S12A,B). indicating that apoptosis is the primary mode of HIF‐induced cell death. These findings demonstrate that HIF, a major homoisoflavanone component of PKF, suppresses CRC cell proliferation and migration primarily by inducing apoptosis and cell cycle arrest.

### 2.5 HIF Induces DNA Damage‐Mediated Parthanatos in CRC Cells

**FIGURE 5:**
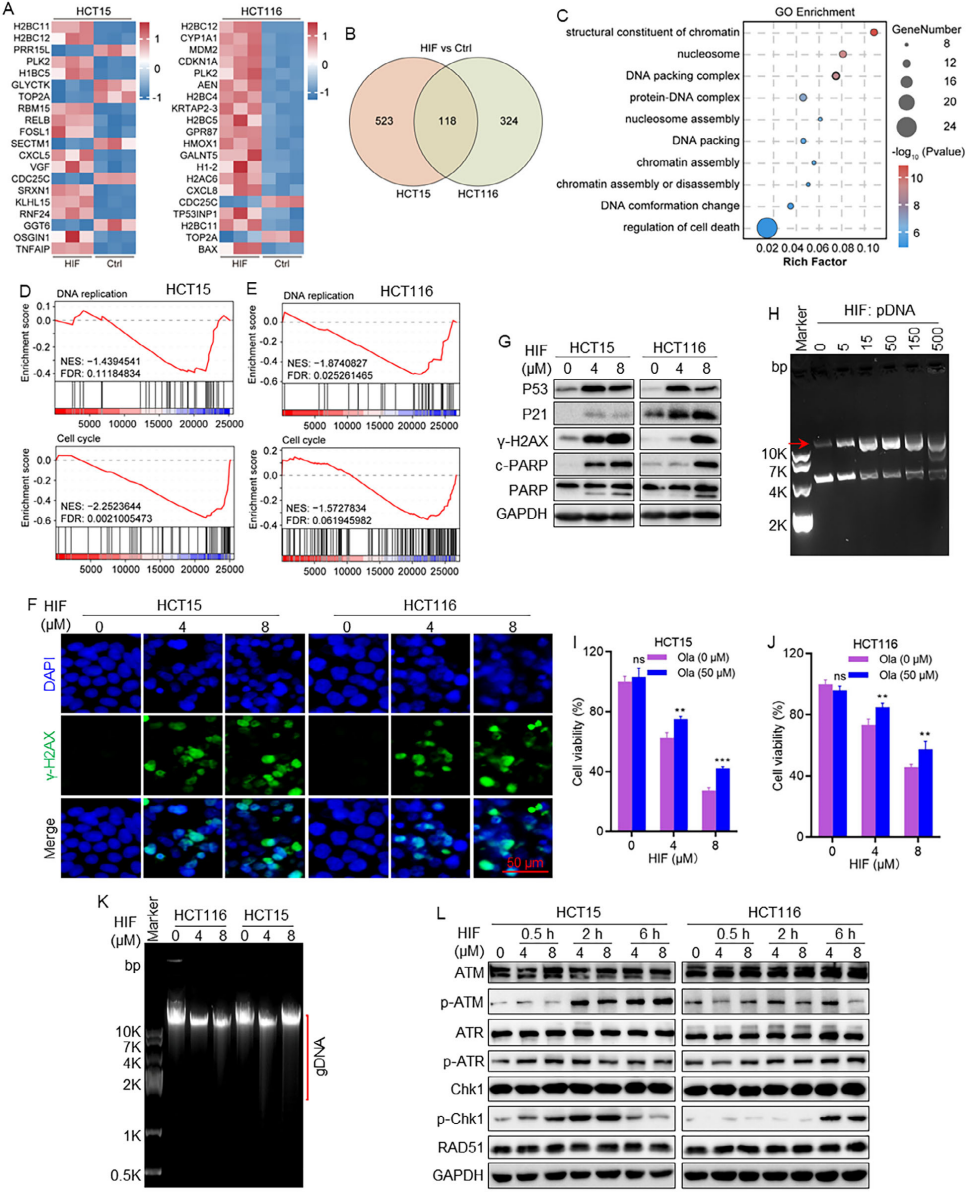
HIF induces DNA damage‐mediated parthanatos in CRC cells. (A) Heatmap of differentially expressed genes in HCT15 and HCT116 cells treated with or without HIF. (B) Venn diagram illustrating overlapping differentially expressed genes between HCT15 and HCT116 cells. (C) GO enrichment analysis of HIF‐regulated genes in HCT15 and HCT116 cells. (D, E) GSEA of genes modulated by HIF in HCT15 and HCT116 cells. (F) Immunofluorescence analysis of γ‐H2AX expression in HCT15 and HCT116 cells following HIF treatment. (G) Expression levels of DNA damage markers (P53, P21, γ‐H2AX, c‐PARP, and PARP). (H) Gel electrophoresis analysis of plasmid DNA treated with HIF. The values represent the mass ratio of PKF to plasmid DNA, indicating that 200 ng of plasmid DNA was incubated with 0, 1, 3, 10, 30, and 100 µg of HIF for 2 h. (I, J) Cell viability assessment of HCT15 and HCT116 cells with HIF or combined with Olaparib (Ola) treatment. (K) Genomic DNA integrity analysis via gel electrophoresis after HIF treatment. (N, O) ROS levels after HIF treatment in HCT15 and HCT116 cells. (L) Time‐course analysis of DNA damage repair‐related proteins, including RAD51 and phosphorylated ATM (p‐ATM), ATR (p‐ATR), and Chk1 (p‐Chk1) in HCT15 and HCT116 cells at 0.5, 2, and 6 h following HIF treatment. ^**^ *p* < 0.01, ^***^ *p* < 0.001, ^****^ *p* < 0.0001; ns, not significant.

To elucidate the mechanism underlying the potent anticancer effects of HIF, transcriptome profiling was performed to identify differentially expressed genes between the HIF‐treated and untreated CRC cells. Heatmap analysis revealed significant upregulation of cell cycle‐related genes such as *CDKN1A*, and chromatin assembly‐related genes including *H2BC11*, *H2B12*, and *H2BC4*, following HIF treatment (Figure 5A). The Venn diagram indicated that 118 differentially expressed genes were shared between HCT15 and HCT116 cells upon HIF exposure (Figure 5B). Gene Ontology (GO) enrichment analysis revealed significant enrichment in chromatin structural components and DNA packing complexes (Figure 5C). Consistently, Gene Set Enrichment Analysis (GSEA) further revealed associations with DNA replication and cell cycle pathways (Figure 5D,E), suggesting a role of HIF in disrupting genomic stability. Immunofluorescence analysis showed elevated levels of γ‐H2AX foci following HIF treatment (Figure 5F), confirming the induction of DNA damage. Based on these findings, we hypothesize that HIF induces DNA damage‐mediated cell death, similar to the mechanism observed for PKF. Western blot analysis showed increased expression of P53, P21, γ‐H2AX, and c‐PARP in both HCT15 and HCT116 cells following HIF treatment (Figure 5G). Notably, DNA gel electrophoresis revealed that HIF led to the formation of nicked or linear species from supercoiled DNA with reduced electrophoretic mobility, indicating HIF‐induced DNA strand breaks (Figure 5H). Functional rescue assays further validated the involvement of a PARP‐dependent cell death pathway. Treatment with Olaparib partially reversed HIF‐induced cell death in both HCT15 and HCT116 cell lines (Figure 5I,J), while combined treatment with Z‐VAD‐FMK produced the most pronounced rescue effect (Figure S12C), suggesting that HIF‐induced cell death involves both apoptosis and PARP‐mediated parthanatos. Consistently, Additionally, HIF treatment led to substantial genomic DNA fragmentation (Figure 5K). While moderate DNA damage can activate DNA damage response pathways to attempt repair, to facilitate repair, excessive or unrepaired lesions ultimately lead to cell death or loss of cellular function [29, 30]. Consistent with this, western blot analysis further revealed increased phosphorylated ATM, ATR, and Chk1, while RAD51 levels remained unchanged in both cell lines following HIF treatment (Figure 5L), indicating activation of DNA damage response signaling.

**FIGURE 6:**
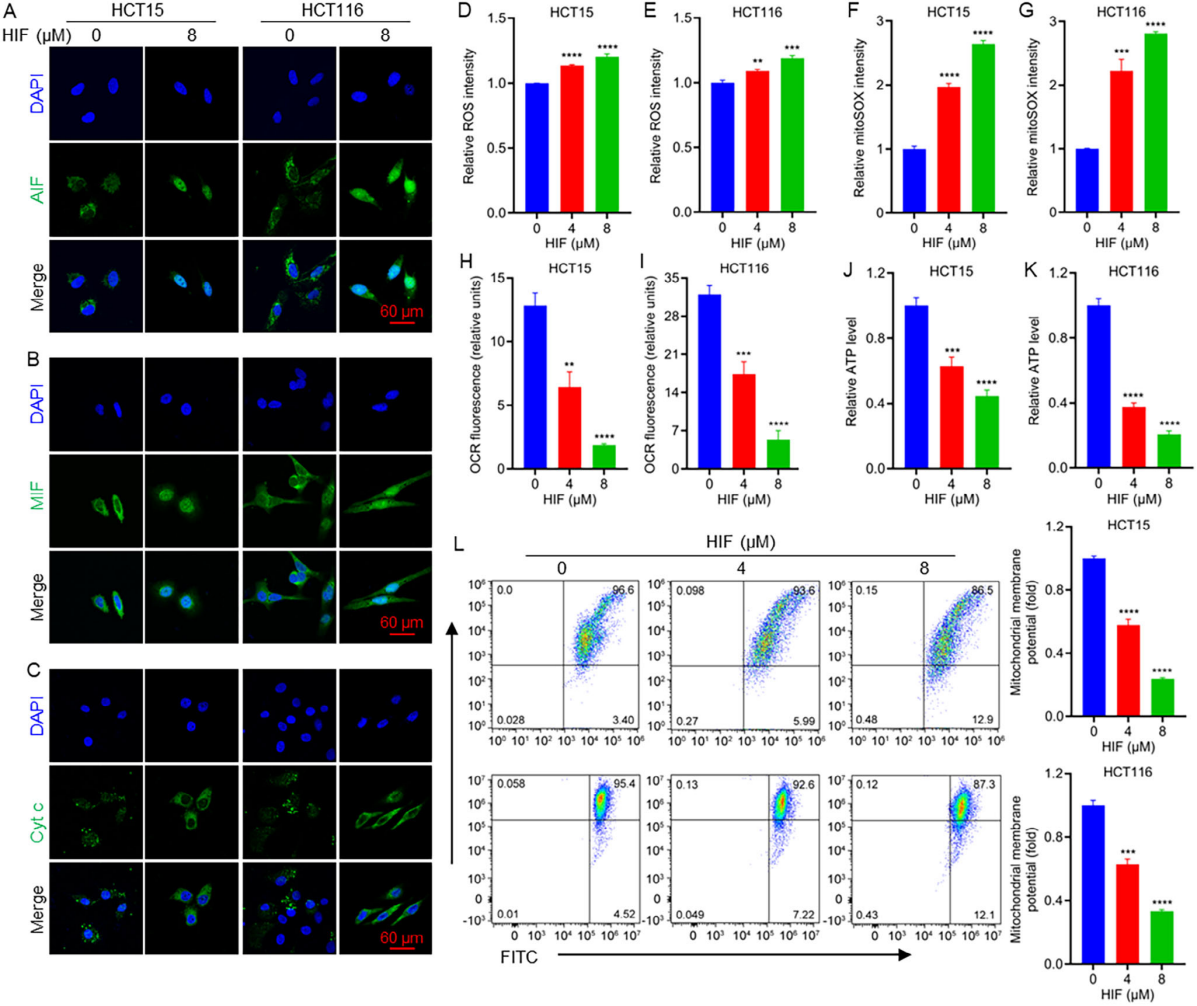
HIF disrupts mitochondrial homeostasis and decreases ATP production. (A–C) Representative immunofluorescence images show the subcellular localization and nuclear translocation of AIF, MIF, and Cytochrome c (Cyt c) in HCT15 and HCT116 cells following 24 h HIF treatment. (D, E) Quantification of intracellular reactive oxygen species (ROS) levels in HCT15 and HCT116 cells, respectively. (F, G) Quantification of mitochondrial ROS intensity using mitoSOX staining in HCT15 and HCT116 cells, respectively. (H, I) Oxygen consumption rate (OCR) fluorescence intensity in HCT15 and HCT116 cells, reflecting changes in mitochondrial respiration. (J, K) Relative intracellular ATP levels in HCT15 and HCT116 cells following HIF treatment. (L) Flow cytometric analysis of mitochondrial membrane potential (Δψm) in HCT15 and HCT116 cells after HIF treatment, assessed using JC‐1 staining. The red‐to‐green fluorescence intensity ratio was used to quantify alterations in membrane potential (right panels). ^**^ *p* < 0.01, ^***^ *p* < 0.001, ^****^ *p* < 0.0001.

Parthanatos is a distinct form of programmed cell death, mechanistically separate from apoptosis, necrosis, and necroptosis, characterized by the overactivation of poly (ADP‐ribose) polymerase‐1 (PARP‐1) in response to severe DNA damage and oxidative stress [31]. PARP‐1 activation leads to mitochondrial release of apoptosis‐inducing factor (AIF), which interacts with macrophage migration inhibitory factor (MIF) in the cytoplasm to form an AIF‐MIF complex that translocates into the nucleus, inducing large‐scale DNA fragmentation [32]. To validate the activation of parthanatos by HIF, we assessed the subcellular localization of AIF and MIF. Mitochondrial fractionation and Western blot analysis confirmed that AIF was released from mitochondria following HIF treatment (Figure S13A,E). Immunofluorescence and Western blotting demonstrated nuclear translocation of both AIF and MIF (Figure 6A,B; Figure S13B–D). Collectively, these results demonstrate that HIF induces extensive DNA damage and promotes parthanatos‐like cell death through PARP‐ and mitochondria‐dependent pathways in CRC cells.

### 2.6 HIF Induces Mitochondrial Apoptosis

Excessive accumulation of reactive oxygen species (ROS) can activate apoptotic pathways [33], facilitating the release of Cytochrome c (Cyt c) from mitochondria into the cytosol [34, 35]. Consistent with this mechanism, HIF treatment induced Cyt c release from mitochondria in both HCT15 and HCT116 cells (Figure 6C; Figure S13E). Flow cytometry analysis further revealed a marked elevation in total and mitochondrial ROS levels following HIF exposure (Figure 6D–G), indicating oxidative stress as an event in HIF‐induced cytotoxicity. To evaluate the functional consequences of ROS accumulation, mitochondrial activity was assessed by measuring the oxygen consumption rate (OCR), ATP contents, and mitochondrial membrane potential (ΔΨm). HIF‐treated cells exhibited a pronounced reduction in OCR (Figure 6H,I), accompanied by significantly decreased cellular ATP levels (Figure 6J,K), reflecting impaired mitochondrial respiration and bioenergetic collapse. The mitochondrial potential measured using the JC‐1 fluorescent probe showed a clear shift from red aggregates to green monomers, indicating loss of membrane potential. Quantitative analysis confirmed a substantial decrease in ΔΨm following HIF treatment (Figure 6L). Collectively, these findings demonstrate that HIF disrupts mitochondrial function, enhances oxidative stress, and compromises energy metabolism in colorectal cancer cells.

**FIGURE 7:**
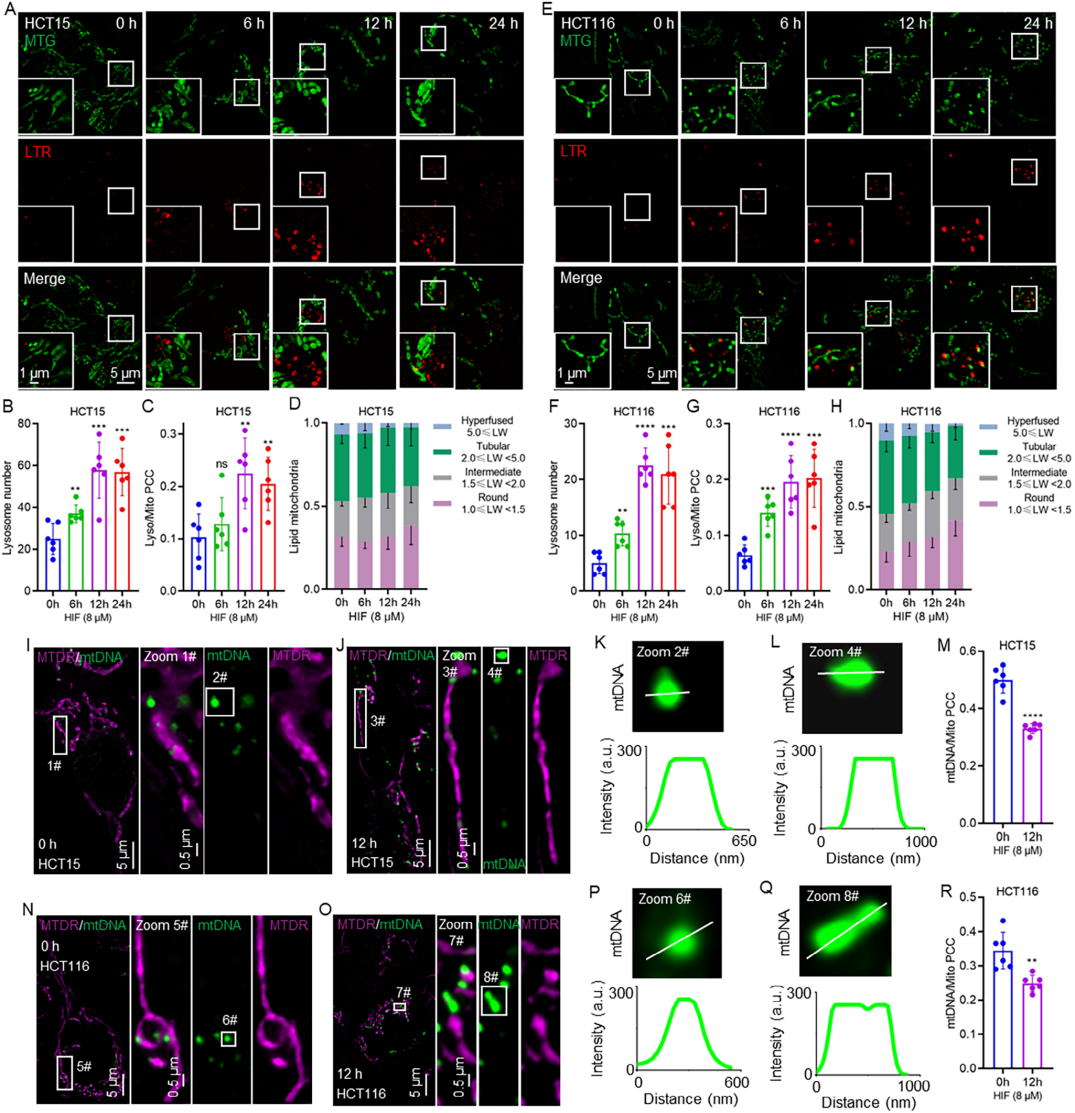
HIF induces mitochondrial apoptosis. (A, E) Structured illumination microscopy (SIM) images of HCT15 and HCT116 cells stained with MitoTracker and LysoTracker following HIF treatment for 0 h, 6 h, 12 h, and 24 h. (B, F) Quantification of lysosome number in HCT15 and HCT116 cells after HIF treatment (n = 6 cells). (C, G) Pearson's colocalization coefficient (PCC) between mitochondria and lysosomes (n = 6 cells). (D, H) Quantitative analysis of mitochondrial fragmentation (n = 10 cells). (I, J, N, O) SIM images of mitochondria and mitochondrial DNA (mtDNA) in HCT15 and HCT116 cells following 12 h of HIF treatment. (K, L, P, Q) Distance‐intensity distribution analysis of mtDNA after HIF treatment. (M, R) Pearson's colocalization coefficient (PCC) analysis of colocalization between mitochondria and mtDNA following HIF treatment (n = 6 cells). ^**^ *p* < 0.01, ^***^ *p* < 0.001, ^****^ *p* < 0.0001; ns, not significant.

Mitochondria are essential organelles for energy metabolism and play a central role in intrinsic apoptosis [36]. Mitochondria membrane damage was observed, and lysosomal activity appeared to contribute to membrane degradation [37, 38]. Lysosomes, as cellular degradation centers and signaling hubs, are critical for maintaining cellular homeostasis and responding to various intra‐ and extracellular cues [39]. Lysosome‐dependent cell death is recognized as one of the regulated cell death subroutines [40]. We hypothesized that lysosome‐mediated mitochondrial apoptosis may contribute to the cytotoxic effects of HIF. Supporting this, lysosomal accumulation was observed in both HCT15 and HCT116 cells upon HIF treatment (Figure 7A,B,E,F; Figure S14). Moreover, fluorescence colocalization analysis revealed a high degree of mitochondria‐lysosome interaction, as indicated by elevated Pearson's colocalization coefficients (PCC) (Figure 7C,G; Figure S15). Mitochondrial membrane permeabilization, a hallmark of apoptosis, is typically followed by mitochondrial fragmentation [41], which increased significantly upon HIF treatment (Figure 7D,H). During mitochondrial membrane permeabilization, mitochondrial DNA (mtDNA) can be released into the cytoplasm, contributing to inflammatory signaling and apoptotic progression [42, 43]. Indeed, HIF treatment led to mtDNA damage, aggregation, and leakage from mitochondria, as evidenced by colocalization analysis (Figure 7I–R). Taken together, these findings indicate that HIF induces mitochondrial apoptosis through multiple converging mechanisms, including ROS accumulation, Cyt c release, mitochondrial membrane depolarization, and lysosome‐mediated mitochondrial apoptosis.

### 2.7 HIF Treatment Suppresses CRC Tumor Growth In Vivo

**FIGURE 8:**
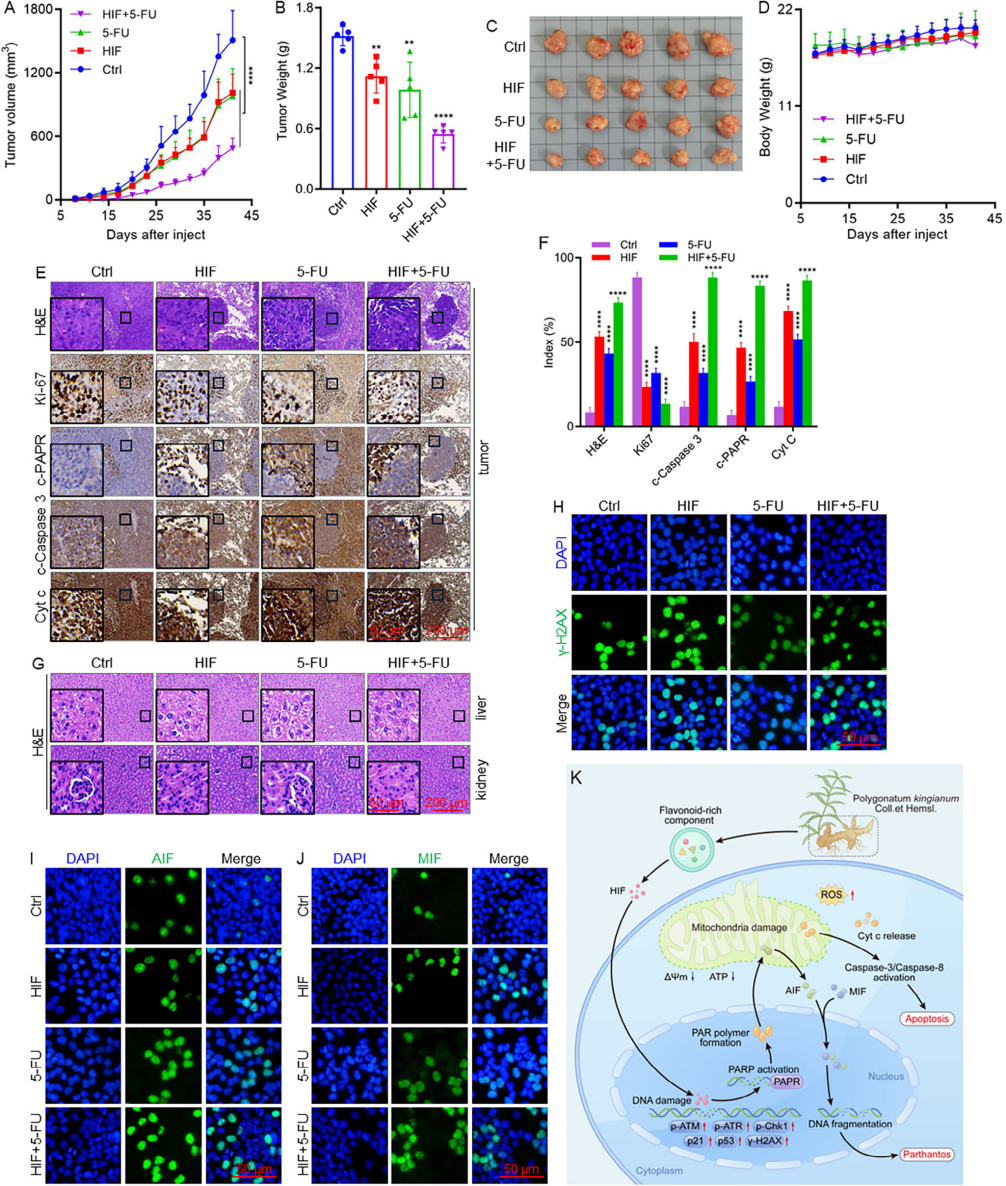
HIF suppresses CRC tumor growth in vivo. (A) Tumor growth in mouse models bearing HCT116 cells treated with HIF, 5‐FU, or the combination. (B) Final tumor weights were measured at the end of treatment across treatment groups. (C) Representative images of excised tumors from each group. (D) Body weight monitoring throughout the treatment period. (E, F) H&E and IHC analysis of tumor tissues for Ki67, Cyt c, c‐Caspase‐3, and c‐PARP, with corresponding quantification. (G) H&E and IHC staining of kidney and liver tissues to evaluate potential organ toxicity. (H–J) Immunofluorescence analysis of γ‐H2AX, AIF, and MIF localization in tumor tissues. Nuclei are stained with DAPI (blue), and target proteins appear in green. (K) Schematic representation illustrating the proposed mechanism by which HIF‐induced DNA damage leads to mitochondrial apoptosis and parthanatos‐mediated colorectal tumor cell death. ^**^ *p* < 0.01, ^****^ *p* < 0.0001.

Following the potent anti‐CRC activity of HIF observed in vitro, its therapeutic efficacy was evaluated using a xenograft mouse model. HCT116 cells were subcutaneously injected into BALB/c nude mice, and after seven days, the mice were randomly assigned to four groups: control, HIF, 5‐FU, and HIF combined with 5‐FU. Treatments were administered daily via intraperitoneal injection. HIF significantly suppresses tumor growth, evidenced by reduced tumor volume and weight compared to the control group. Furthermore, HIF enhanced the antitumor efficacy of 5‐FU in the combination group (Figure 8A–C). The body weight of HIF‐treated mice remained stable, suggesting minimal systemic toxicity (Figure 8D). H&E staining of liver and kidney tissues revealed no pathological changes, indicating that HIF did not induce organ toxicity (Figure 8G). To elucidate the mechanism of HIF‐mediated tumor suppression, immunohistochemical analysis was conducted on tumor tissues. HIF treatment substantially decreased the expression of proliferation marker Ki67 and increased the levels of c‐PARP, c‐Caspase‐3, and Cyt c, indicating the induction of apoptotic cell death (Figure 8E,F). Immunofluorescence analysis further revealed elevated γ‐H2AX expression in tumor tissues, confirming HIF‐induced DNA damage (Figure 8H). To determine whether HIF‐induced tumor suppression involves parthanatos, the nuclear translocation of AIF and MIF was examined. Co‐localization of AIF or MIF with nuclear signals indicated their translocation from the cytoplasm to the nucleus. HIF treatment significantly promoted nuclear translocation of both AIF and MIF, with stronger fluorescence intensity observed in HIF‐treated tumors compared to the control and 5‐FU groups (Figure 8I,J). These findings collectively demonstrate that HIF suppresses CRC progression by inducing both mitochondrial apoptosis and parthanatos.

## 3 Discussion

Colorectal cancer represents the third most lethal malignancy worldwide, with a five‐year survival rate of approximately 64%, which declines to 12% in metastatic cases [2]. Despite advances in chemotherapy and targeted therapies, clinical outcomes remain suboptimal, emphasizing the urgent need for novel, safer, and more effective therapeutic strategies [44, 45]. Herbal medicine has garnered increasing attention as an adjunct or alternative approach, supported by extensive preclinical evidence demonstrating its anticancer potential [46, 47]. Among herbal‐derived phytochemicals, flavonoids constitute a diverse class of bioactive compounds exhibiting potent anticancer activity. Representative flavonoids, including the flavanol epigallocatechin‐3‐gallate, the flavonol quercetin, and the isoflavone genistein, exert anticancer effects through cell cycle arrest and apoptosis induction [48]. In this study, we revealed that the total flavonoids isolated from *Polygonatum kingianum* suppressed CRC cell proliferation and tumor growth primarily through the induction of cell cycle arrest and apoptosis. PKF triggered robust DNA damage, as evidenced by upregulation of γ‐H2AX, p53, and cleaved‐PARP, while PARP inhibitor Olaparib partially restored cell viability, suggesting a PARP‐dependent cell death mechanism. Guided by bioactivity‐directed fractionation, a key bioactive homoisoflavanone (HIF) was subsequently purified and structurally confirmed vis LC‐MS and NMR analyses. HIF reproduced the anticancer effects of PKF, causing pronounced DNA damage characterized by the accumulation of γ‐H2AX, activation of P53, and the appearance of cleaved‐PARP, hallmarks of DNA damage‐associated apoptosis.

DNA damage is a fundamental cellular response to diverse stress stimuli and can manifest in multiple forms, including base modifications, crosslinking, single‐ and double‐strand breaks. Each type of lesion activates distinct DNA damage response pathways that detect, signal, and repair the damage. However, when the damage is too severe or if repair mechanisms fail, it can lead to cell death or impair cellular function [28, 49, 50]. However, when the damage is excessive or the repair mechanisms are overwhelmed, cells may undergo irreversible dysfunction or programmed death [51]. HIF treatment markedly enhanced phosphorylation of ATM, ATR, and Chk1, key upstream DDR kinases, indicating activation of the canonical checkpoint signaling. However, the expression of RAD51, a central recombinase for homologous recombination (HR) repair, remained unchanged, suggesting that downstream HR repair was defective. This disconnect can create a state of “unresolvable” DNA damage, driving prolonged checkpoint activation and apoptosis [52]. Transcriptomic analyses further supported this mechanism, revealing enrichment of cell cycle and DNA replication pathways following HIF treatment. These findings suggest HIF induces a DDR‐related stress and apoptosis response that culminates in apoptosis.

PARP1 functions as a nuclear enzyme regulating diverse cellular processes through PARylation, including DNA repair, chromatin remodeling, and transcription [53]. Upon sensing DNA single‐ or double‐strand breaks, PARP1 catalyzes the synthesis of poly(ADP‐ribose) (PAR) polymers using NAD^+^ as a substrate, facilitating recruitment of downstream repair factors [54]. Although PARP inhibitors function by preventing single‐strand break repair, affecting replication fork progression, particularly benefiting patients with BRCA1/2‐wild‐type tumors [55]. However, excessive PARP activation can paradoxically trigger a distinct caspase‐independent cell death termed parthanatos. Overactivation of PARP leads to massive PAR accumulation, NAD^+^ depletion, and nuclear‐to‐cytoplasmic export of PAR polymers, which promote apoptosis‐inducing factor (AIF) release from mitochondria and its interaction with macrophage migration inhibitory factor (MIF), The resulting AIF‐MIF complex translocates to the nucleus, causing large‐scale DNA fragmentation and cell death [56, 57]. Consistent with this mechanism, HIF treatment induced mitochondrial release and nuclear translocation of both AIF and MIF. Moreover, PARP inhibition significantly enhanced cell survival in HIF‐treated CRC cells, confirming that HIF suppresses CRC tumor growth through PARP‐dependent parthanatos.

Mitochondria play a pivotal role in cellular energy metabolism and intrinsic apoptosis, integrating signals from ROS accumulation, metabolic stress, and organelle crosstalk [58, 59]. Mitochondria‐dependent apoptosis is primarily associated with mitochondrial membrane permeabilization, which facilitates cytochrome c release and subsequent caspase activation. In addition to cytochrome c, other mitochondrial proteins, including AIF, function as critical regulators of cell death, mediating caspase‐independent parthanatos‐like cell death [60, 61]. HIF exposure led to severe mitochondrial dysfunction, characterized by excessive mitochondrial ROS production, decreased oxygen‐consumption rate (OCR), reduced ATP generation, and depolarization of the mitochondrial membrane potential (ΔΨm). These alterations reflect compromised oxidative phosphorylation and loss of mitochondrial integrity. Consequently, cytochrome c was released into the cytosol, activating the caspase cascade and initiating mitochondrial apoptosis. Accompanying these events, mitochondrial DNA (mtDNA) damage and release were detected, together with enhanced mitochondria‐lysosome contact, implicating lysosome‐mediated mitochondrial degradation as a contributing mechanism. Collectively, these findings delineate a dual mechanism of mitochondrial apoptosis activation and parthanatos, ultimately leading to cell death.

Although the precise molecular targets of HIF remain unidentified, its conjugated benzopyran ring scaffold and phenolic hydroxyl groups suggest potential for DNA intercalation or redox‐mediated genotoxicity. Similar structural motifs in flavonoids have been shown to bind DNA or modulate topoisomerase activity [62]. The observed increase in oxidative stress and accumulation of γ‐H2AX further indicates that HIF may induce ROS production, thereby enhancing DNA strand breaks and replication stress. Future investigations will employ targeted proteomic and chemical biology approaches, including drug affinity responsive target stability, cellular thermal shift assays, and molecular docking, to identify HIF‐interacting proteins, such as PARP1 and topoisomerase II, which are involved in DNA repair and replication. Therapeutically, HIF‐induced DNA damage may be particularly effective against colorectal cancer with mismatch repair or homologous recombination deficiencies and could overcome 5‐FU resistance by impairing DNA synthesis and repair. Thus, HIF represents a promising flavonoid‐derived agent that triggers apoptosis and parthanatos to enhance treatment efficacy in intractable colorectal cancer. In summary, this study identifies HIF as a key bioactive homoisoflavanone isolated from *Polygonatum kingianum* that exerts potent anti‐CRC effects in vitro and in vivo. HIF induces DNA damage‐driven apoptosis and PARP‐dependent parthanatos, accompanied by profound mitochondrial dysfunction and oxidative stress (Figure 8K). These findings highlight *Polygonatum kingianum* as a valuable source of anticancer flavonoids and underscore the potential of natural products as a promising strategy for colorectal cancer therapy.

## 4 Experimental Section

### 4.1 Isolation and Structural Characterization of Compounds

Rhizomes of Polygonatum *kingianum* were acquired from Yipuyuan Huangjing Technology Co., Ltd. (Xinhua, Hunan, China). Flavonoid components were isolated following conventional phytochemical procedures, and multiple major compounds were successfully purified. The structures of N‐feruloyloctopamine, N‐p‐trans‐coumaroyltyramine, N‐trans‐feruloyltyramine, and Disporopsin were identified through comparison with authentic standards, including N‐feruloyloctopamine (66648‐44‐0, Targetmol), N‐p‐trans‐coumaroyltyramine (36417‐86‐4, Targetmol), N‐trans‐feruloyltyramine (66648‐43‐9, Targetmol), and Disporopsin (1430334‐05‐6, BioBioPha) and confirmed by LC‐MS analysis. The structure of 4’,5,7‐trihydroxy‐6,8‐dimethylhomoisoflavanone (HIF) was further characterized by LC‐MS (reference standard: 189264‐18‐4, BLDpham) and NMR spectroscopy. UPLC‐QTOF‐MS (Agilent Technologies) analysis was performed an Acquity BEH C18 column (2.1 × 100 mm, 1.7 µm). The mobile phase consisted of a gradient of acetonitrile‐water (0–2 min, 5%; 2–12 min, 5–90%; 12–15 min, 90%), at a flow rate 0.4 mL/min, 30 °C, with a 1 µL injection volume. Detection was performed on an Agilent 6545 Q‐TOF mass spectrometer in negative ion mode with Dual AJS ESI settings: gas temperature 320 °C, drying gas 8 L/min, nebulizer 35 psi, sheath gas 350 °C/11 L/min, capillary voltage 3500 V, nozzle voltage 1000 V, fragmentor voltage 75 V, skimmer voltage 65 V, *m/z* 100–1000, collision energy of 20/40/60 eV.

All NMR spectra were acquired on a Bruker Avance Neo 600 MHz spectrometer equipped with a DCH CryoProbe using DMSO‐d6 as solvent and internal reference. Parameters for conventional NMR experiments are detailed in Table S1. Data were processed using Topspin (v4.5.0) or MestReNova (v9.0.1), and peak assignments were confirmed by comparison with published data [63, 64]. Due to signal overlap of HIF in the 2.5–4.4 ppm region in the ^1^H NMR spectrum (Figures S4 and S5), selective 1D TOCSY experiments were performed to resolve overlapping resonances and clarify splitting patterns (Figure S6). Most key resonances were successfully assigned and demonstrated full consistency with the proposed structure (Figures S7–S11). Final chemical shift assignments are summarized in Table S2.

### 4.2 Cell Lines and Culture

All cell lines were obtained from the National Collection of Authenticated Cell Cultures. HCT116 cells were cultured in McCoy's 5A (Modified) medium (16600082, Gibco), while HCT15, NCM460, and LO2 cells were maintained in RPMI 1640 medium (11875093, Gibco). All media were supplemented with 10% fetal bovine serum (FBS; A5670801, Gibco) and 1% penicillin‐streptomycin (15140122, Gibco). Cells were incubated at 37°C in a humidified atmosphere containing 5% CO_2_.

### 4.3 Animal Experiments

Specific‐pathogen‐free (SPF) female BALB/c nude mice (6–8 weeks, 18–22 g) were purchased from GemPharmatech Co., Ltd. (SCXK(SU) 2023‐0009) and maintained under SPF conditions (22 ± 2°C, 60% ± 10% humidity, a 12 h light/dark cycle) at the Laboratory Animal Research Center of Nanchang University (SYXK(GAN)2021‐0004). All animal procedures were approved by the Institutional Animal Care and Use Committee of Nanchang University (NCULAE‐20240822002) and complied with institutional and national guidelines. For xenograft establishment, HCT116 cells (1×10^6^) were subcutaneously injected into the flanks of BALB/c nude mice. When tumor volume reached approximately 50 mm^3^ (1/2 × length × width^2^), mice were randomized into six groups (n = 5): control, L‐PKF (10 mg/kg), M‐PKF (30 mg/kg), H‐PKF (90 mg/kg), 5‐FU (10 mg/kg, S1209, Selleck), and a combination of H‐PKF (90 mg/kg) with 5‐FU (10 mg/kg). All treatments were administered through intraperitoneally once daily. Tumor size and body weight were monitored every 3 days. At study completion, tumors were excised, weighed, and fixed in 4% paraformaldehyde for histological and immunohistochemical analyses. Liver and kidney tissues were also collected for H&E staining to assess systemic toxicity. To evaluate HIF efficacy, tumor‐bearing mice were assigned to four groups (n = 5): control, HIF (10 mg/kg), 5‐FU (10 mg/kg), and a combination of HIF (10 mg/kg) with 5‐FU (10 mg/kg). All groups received intraperitoneal injections under identical conditions.

### 4.4 Cell Viability Assay

Cell viability assessment using Cell Counting Kit‐8 (CCK‐8, C0005, TargetMol). Cells were seeded in 96‐well plates at 5×10^3^ cells per well and treated with PKF or HIF for 48 h, either alone or in combination with Z‐VAD‐FMK (S7023, Selleck), Necrostatin‐1 (Nec‐1, S8037, Selleck), Ferrostatin‐1 (Fer‐1, S7243, Selleck), and Olaparib (S1060, Selleck). Following treatment, 10 µL of CCK‐8 reagents were added to each well and incubated for 1 h at 37°C, and absorbance was recorded at 450 nm.

### 4.5 Colony Formation Assay

Cells were seeded in 6‐well plates at 1.2×10^3^ cells per well and treated with PKF or HIF for 7 days, with medium replaced as needed. Colonies were fixed with 4% paraformaldehyde for 10 min, and stained with 0.5% crystal violet, washed, and counted.

### 4.6 Cell Migration and Invasion Assay

200 µL of cell suspension with 5×10^5^ cells per mL in serum‐free media was seeded into the upper chamber of 8‐µm Transwell insert (3422, Corning). The lower chamber contained 600 µL of complete medium with 10% FBS as a chemoattractant. Following 48 h incubation at 37°C, non‐migrated cells were removed, and migrated cells were fixed with 4% paraformaldehyde for 15 min, stained with 0.5% crystal violet, and counted under light microscopy.

### 4.7 Apoptosis Detection

HCT116 and HCT15 cells were treated with PKF or HIF for 24 h, harvested, and stained with Annexin V‐FITC (FA101‐02, TransGen) for 15 min in the dark, followed by propidium iodide staining (F10797, Invitrogen) for 5 min. Samples were analyzed using a CytoFLEX S flow cytometer (Beckman Coulter), and data were processed using FlowJo.

### 4.8 Cell Cycle Analysis

HCT116 and HCT15 cells were treated with PKF or HIF for 24 h, harvested, and fixed with pre‐chilled 75% ethanol at 4°C overnight. The cells were washed with PBS and stained with propidium iodide (F10797, Invitrogen) for 1 h at 4°C in the dark. DNA content analysis was performed using a CytoFLEX S flow cytometer (Beckman Coulter), and cell cycle distribution was analyzed using FlowJo.

### 4.9 Western Blotting Analysis

Cells and tumor tissues were lysed in RIPA buffer supplemented with protease inhibitor cocktail (C0001, TargetMol) and phosphatase inhibitor cocktail (C0004, TargetMol). Protein concentrations were determined by BCA assay (P0012, Beyotime). Equal protein amounts were separated by SDS‐PAGE and subsequently transferred to PVDF membranes. The membranes were then blocked with a 5% non‐fat milk TBST (TBS containing 0.1% Tween‐20) for 1 h at room temperature and incubated at 4°C for overnight with primary antibodies against the following proteins: cleaved Caspase 3 (25128‐1‐AP, Proteintech), cleaved Caspase 8 (9496, CST), PARP (9542, CST), cleaved PARP (9541, CST), P21 (2947, CST), P53 (2527, CST), Cyclin A2 (91500, CST), Cyclin D1 (2978, CST), CDK1 (bs‐1341R, Bioss), CDK2 (bs‐10726R, Bioss), CDK7 (bs‐0569R, Bioss), AKT (4691, CST), p‐AKT (4060, CST), ERK (9102, CST), p‐ERK (9101, CST), MEK (9126, CST), p‐MEK (9154, CST), ATR (13934, CST), p‐ATR (2853, CST), Chk1 (2360, CST), p‐Chk1 (2348, CST), RAD51 (14961‐1‐AP, Proteintech), ATM (27156‐1‐AP, Proteintech), p‐ATM (5883, CST), γ‐H2AX (9718, CST), AIF (5318, CST), MIF (75038, CST), Cyt c (10993‐1‐AP, Proteintech), Lamin B (66095‐1‐Ig, Proteintech), VDAC1 (81538‐1‐RR, Proteintech) and GAPDH (60004‐1‐Ig, Proteintech). Following primary antibody incubation, the membranes were washed again with TBST and incubated with HRP‐conjugated goat anti‐mouse IgG (H+L) (SA00001‐1, Proteintech) and goat anti‐rabbit IgG (H+L) (SA00001‐2, Proteintech) for 1 h at room temperature. Protein bands were visualized using SuperSignal West Atto Ultimate Sensitivity Substrate (A38556, Thermo Scientific), captured using the ChemiDoc MP Imaging System (Bio‐Rad), and quantified using ImageJ software (National Institutes of Health).

### 4.10 RNA‐seq Analysis

Total RNA was extracted from CRC cells treated with PKF or HIF for three consecutive days using TRIzoL Reagent (15596026CN, Invitrogen). RNA quality and integrity were evaluated using the Bioanalyzer 2100 system (Agilent Technologies). Strand‐specific RNA‐seq libraries were prepared using the TruSeq Stranded Total RNA Library Prep Kit (Illumina, San Diego, USA) according to the manufacturer's protocol. The libraries were sequenced on the Illumina NovaSeq 6000 platform (LC‐Bio Technologies, China), and bioinformatic analyses were conducted using the OmicStudio tools available at https://www.omicstudio.cn/tool.

### 4.11 Hematoxylin and Eosin (H&E), Immunohistochemistry (IHC), and Immunofluorescence (IF) Staining

Paraffin‐embedded tumor tissues were sectioned into 5 µm slices for H&E staining. For IHC staining, the sections underwent dewaxing in xylene, rehydration through gradient ethanol, and permeabilization with 0.1% Triton X‐100. Following antigen retrieval, nonspecific binding was blocked using appropriate blocking solutions. The tissue sections were subsequently incubated with primary antibodies against cleaved Caspase 3 (1:1000, GB115733‐100, Servicebio), cleaved PARP (1:1000, GB111503, Servicebio), Cyt c (1:800, AF2047, Beyotime), HGMB1 (1:1000, GB11103‐100, Servicebio), and Ki‐67 (MAB‐0672, MXB), followed by incubation with HRP‐conjugated secondary antibodies (SD3100, Celnovte) for 20 min at 37°C in darkness. Visualization of stained slices was performed using a BX 53 upright microscope (Olympus, Tokyo, Japan).

For immunofluorescence staining, paraffin‐embedded tissues were sectioned into 2 µm slices, dewaxed, rehydrated, and permeabilized with 0.1% Triton X‐100. After blocking nonspecific binding with 10% bovine serum albumin (BSA, BS114‐100 g, Biosharp), tissue autofluorescence was quenched using an autofluorescence quenching reagent (G1221‐5ML, Servicebio). The sections underwent overnight incubation at 4°C with primary antibodies against AIF (1:100, F0268, Selleck), MIF (1:100, 75038, CST), and γ‐H2AX (1:100, 9718, CST). Following washing, Alexa Fluor 488‐labeled goat anti‐rabbit IgG (H+L) (A0423, Beyotime) secondary antibodies were applied for 1 h at 37°C, followed by DAPI nuclear counterstaining for 5 min. Image capture and analysis were performed using the Leica HCS A imaging system.

### 4.12 Confocal Immunofluorescence Microscopy

HCT116 and HCT15 cells (1×10^4^ per well) received treatment with 8 µM HIF for 24 h. Subsequently, cells underwent fixation with 4% paraformaldehyde for 30 min, permeabilization with 0.1% Triton X‐100 for 5 min and blocking with 5% BSA for 1 h at room temperature. The cells were then incubated overnight at 4°C with primary antibodies against AIF (1:100, F0268, Selleck), MIF (1:100, 75038, CST), Cyt c (1:200, AF2047, Beyotime), and γ‐H2AX (1:200, 9718, CST). The following day, Alexa Fluor 488‐conjugated goat anti‐rabbit IgG (H+L) (A0423, Beyotime) secondary antibodies were applied for 1 h at 37°C, followed by DAPI staining for 5 min. Image acquisition was performed using an Olympus Fluoview FV3000 confocal laser scanning microscope.

### 4.13 Reactive Oxygen Species (ROS) Detection

For total cellular ROS, cells were plated in 6‐well plates, treated with HIF for 24 h, and incubated with 10 µM DCFH‐DA (HY‐D0940, MCE) at 37°C for 30 min. Mitochondrial ROS levels were assessed using the MitoSOX Red Kit (HY‐D1055, MCE) according to the manufacturer's instructions. After staining, cells were resuspended in Dulbecco's Phosphate‐Buffered Saline (DPBS) and immediately analyzed using a CytoFLEX S flow cytometer (Beckman Coulter). ROS levels were quantified based on DCF fluorescence (cellular ROS) or MitoSOX Red fluorescence (mitochondrial ROS).

### 4.14 Oxygen Consumption Rate (OCR)

OCR was measured using the OCR Fluorometric Assay Kit (E‐BC‐F068, Elabscience) according to the manufacturer's protocol. The microplate was placed in a multifunctional microplate reader set at 37°C in dynamic reading mode. The excitation and emission wavelengths were set at 405 nm and 675 nm, respectively. Fluorescence intensity was recorded every 2 min for 180 min. OCR was calculated based on the slope of the fluorescence intensity curve over time.

### 4.15 ATP Content Detection

ATP levels were measured using the ATP Assay Kit (HY‐K0314, MCE) following the manufacturer's instructions. Cells were seeded in 96‐well plates and treated with HIF for 24 h. After treatment, cells were lysed, and the supernatants were collected for analysis. ATP detection working solution was added to each well and briefly incubated in the dark. Luminescence was measured using a cell imaging multimode reader (BioTek Cytation 5, Agilent). ATP concentrations were determined using a standard curve and expressed as relative light units as indicated.

### 4.16 Mitochondrial Membrane Potential Detection

Cells were seeded in 6‐well plates and treated with HIF for 24 h. JC‐1 dye (HY‐15534, MCE) was added to the medium at a final concentration of 10 µg/mL and incubated at 37°C for 20 min. Following incubation, the medium was replaced, and mitochondrial membrane potential was assessed using a CytoFLEX S flow cytometer (Beckman Coulter). High membrane potential was indicated by J‐aggregates (red fluorescence, 525 nm/590 nm), while low membrane potential was indicated by JC‐1 monomers (green fluorescence, 490 nm/530 nm). The red/green fluorescence ratio was used to quantify changes in membrane potential.

### 4.17 Structure Illumination Microscopy

HCT15 and HCT116 cells (1× 10^5^ per well) were seeded into 35 mm glass‐bottom dishes and incubated for 24 h at 37°C. The cells then were then treated with HIF for 6, 12, or 24 h. Post‐treatment, cells were incubated with MitoTracker Green FM (MTG, M7514, Invitrogen) and LysoTracker Red DND‐99 (LTR, L7528, Invitrogen) for 30 min at 37°C, followed by PBS washing. For mitochondrial DNA and membrane visualization, cells underwent HIF treatment in phenol red‐free RPMI 1640 medium (11835030, Gibco) with 10% FBS for 12 h, followed by staining with MitoTracker Deep Red FM (MTDR, M22426, Invitrogen) for 30 min and the Quant‐iT PicoGreen dsDNA Kit (P11496, Invitrogen) for 18 min at 37°C. After PBS washing, images were acquired using an Elyra 7 microscope equipped with a 63 ×/1.49 NA oil‐immersion objective (Carl Zeiss, Inc.), and analyzed using ZEN 2012 software (Carl Zeiss, Inc.) and ImageJ software (National Institutes of Health).

### 4.18 Isolation of Nuclear and Mitochondrial Proteins

Nuclear and cytoplasmic proteins were extracted utilizing the NE‐PER Nuclear and Cytoplasmic Extraction Reagents (78833, Thermo Scientific) according to the manufacturer's protocol. Mitochondrial proteins were isolated utilizing the Cell Mitochondria Isolation Kit (C3601, Beyotime). A quantity of 30 µg of total protein per sample underwent separation by 12% SDS‐PAGE and transfer onto a PVDF membrane for subsequent analysis. Following blocking with 5% non‐fat milk, the membranes underwent incubation with appropriate primary antibodies and development using standard western blotting procedures.

### 4.19 DNA Gel Electrophoresis

For genomic DNA fragmentation analysis, HCT15 and HCT116 cells underwent HIF treatment for 24 h and subsequent harvesting. Genomic DNA extraction was performed using the Genomic DNA Kit (9765, Takara) following the manufacturer's protocol. For the DNA binding assay, 200 ng of plasmid DNA underwent incubation with varying concentrations of PKF (0, 2, 6, 20, 60, and 200 µg) or HIF (0, 1, 3, 10, 30, and 100 µg) for 2 h at 37°C. Genomic DNA samples and DNA‐compounds complex underwent separation on a 1% agarose gel, and DNA fragmentation visualization was performed using a ChemiDoc MP Imaging System (Bio‐Rad).

### 4.20 Statistical Analysis

All data (unless otherwise indicated) are represented as the mean ± standard deviation (SD) from at least three independent experiments. Statistical analyses were performed using GraphPad Prism version 8.0. Differences between the two groups were determined using Student's *t*‐test, while comparisons among multiple groups were evaluated using one‐way or two‐way ANOVA. A *p*‐value < 0.05 was considered statistically significant.

## Author Contributions

H.F. performed most of the experiments, with assistance from P.Y. and Y.J. H.Z. conducted the immunohistochemical and immunofluorescence analysis. P.Z. and J.L. contributed to data acquisition and analysis. H.J., Y.L. and G.C. were responsible for the isolation and structural characterization of compounds. Y.C. X.L. and A.L. conceived, designed, and supervised the study. Y.C. and H.F. drafted the manuscript with input from all authors. Y.C., X.L., A.L., and Z.C. revised and edited the manuscript. All authors have read and approved the final version of the manuscript.

## Conflicts of Interest

The authors declare no conflicts of interest.

## Supporting information




**Supporting File**: advs74107‐sup‐0001‐SuppMat.docx.

## Data Availability

The data that support the findings of this study are available from the corresponding author upon reasonable request.
