# Supplementary material for: Homoisoflavanone Delays Colorectal Cancer Progression via DNA Damage‐Induced Mitochondrial Apoptosis and Parthanatos‐Like Cell Death
Source: Adv Sci (Weinh). 2026 Jan 28;13(19):e11406. doi: 10.1002/advs.202511406 (PMC13045231; doi:10.1002/advs.202511406)
Supplement: Supplementary file 1 — Supporting File: advs74107‐sup‐0001‐SuppMat.docx. [file ADVS-13-e11406-s001.docx]

**
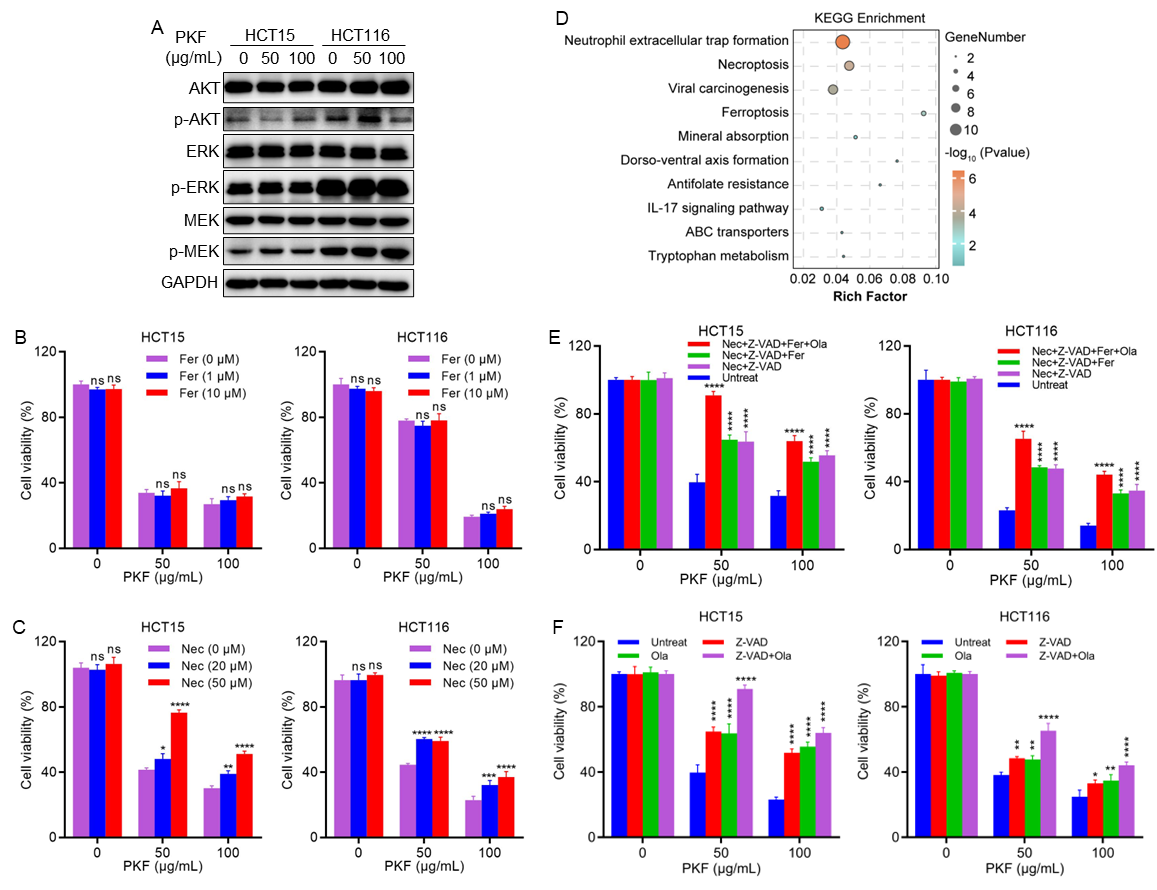
**

**Figure S1**. Effects of PKF and cell death pathway inhibitors on cell viability and pathway regulation in HCT15 and HCT116 cells. A) Protein expression levels of phosphorylated AKT (p-AKT), ERK (p-ERK), and MEK (p-MEK) in HCT15 and HCT116 cells following PKF treatment for 24 h. B, C) Cell viability of HCT15 and HCT116 cells following treatment with PKF alone or in combination with Ferrostatin-1 (Fer) and Necrostatin-1 (Nec). D) KEGG pathway enrichment modulated by PKF in HCT15 and HCT116 cells. E) Cell viability of HCT15 and HCT116 cells following treatment with PKF in combination with Nec, Z-VAD-FMK (Z-VAD), Fer, and Olaparib (Ola). F) Cell viability of HCT15 and HCT116 cells treated with HIF alone or in combination with Z-VAD-FMK (Z-VAD), Olaparib (Ola), or both. * P < 0.05, ** P < 0.01, *** P < 0.001, **** P < 0.0001; ns, not significant.

**
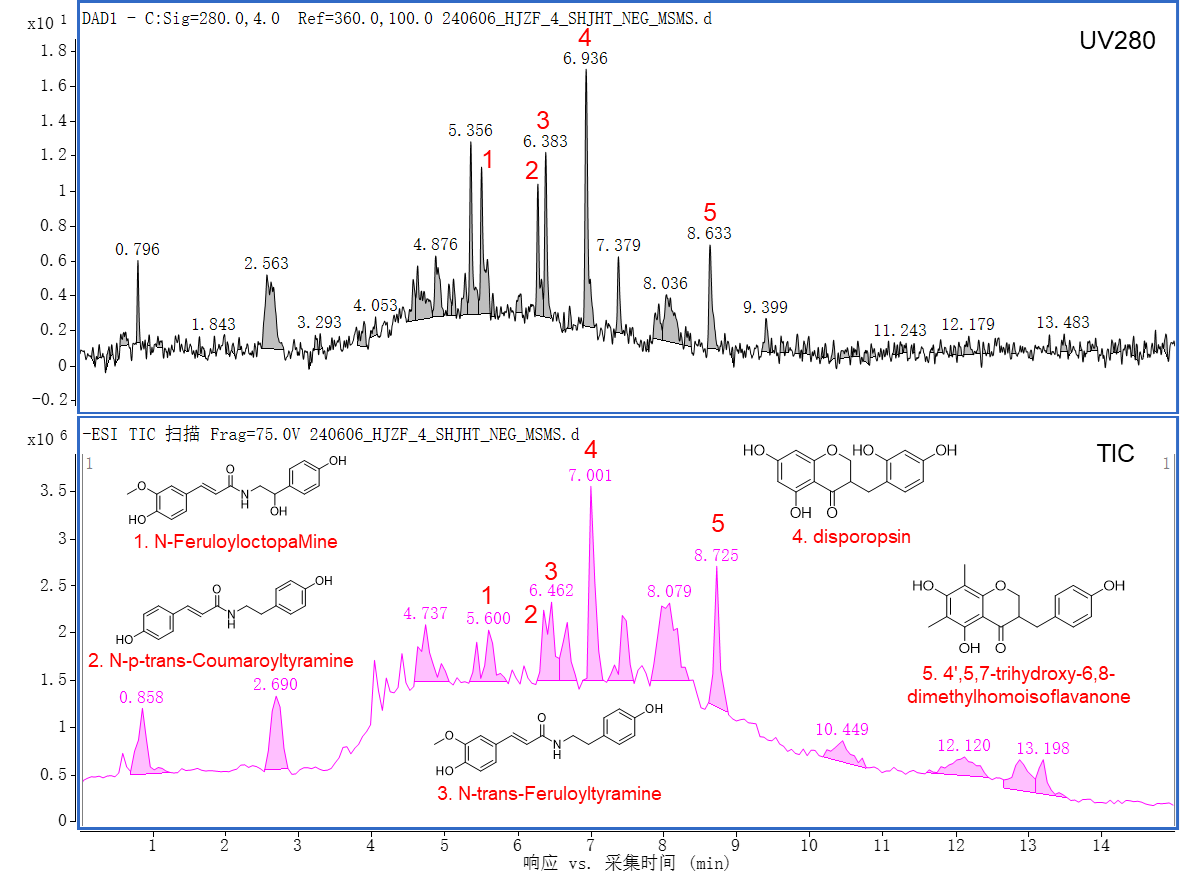
**

**Figure S2**. HPLC chromatogram of PKF.

**
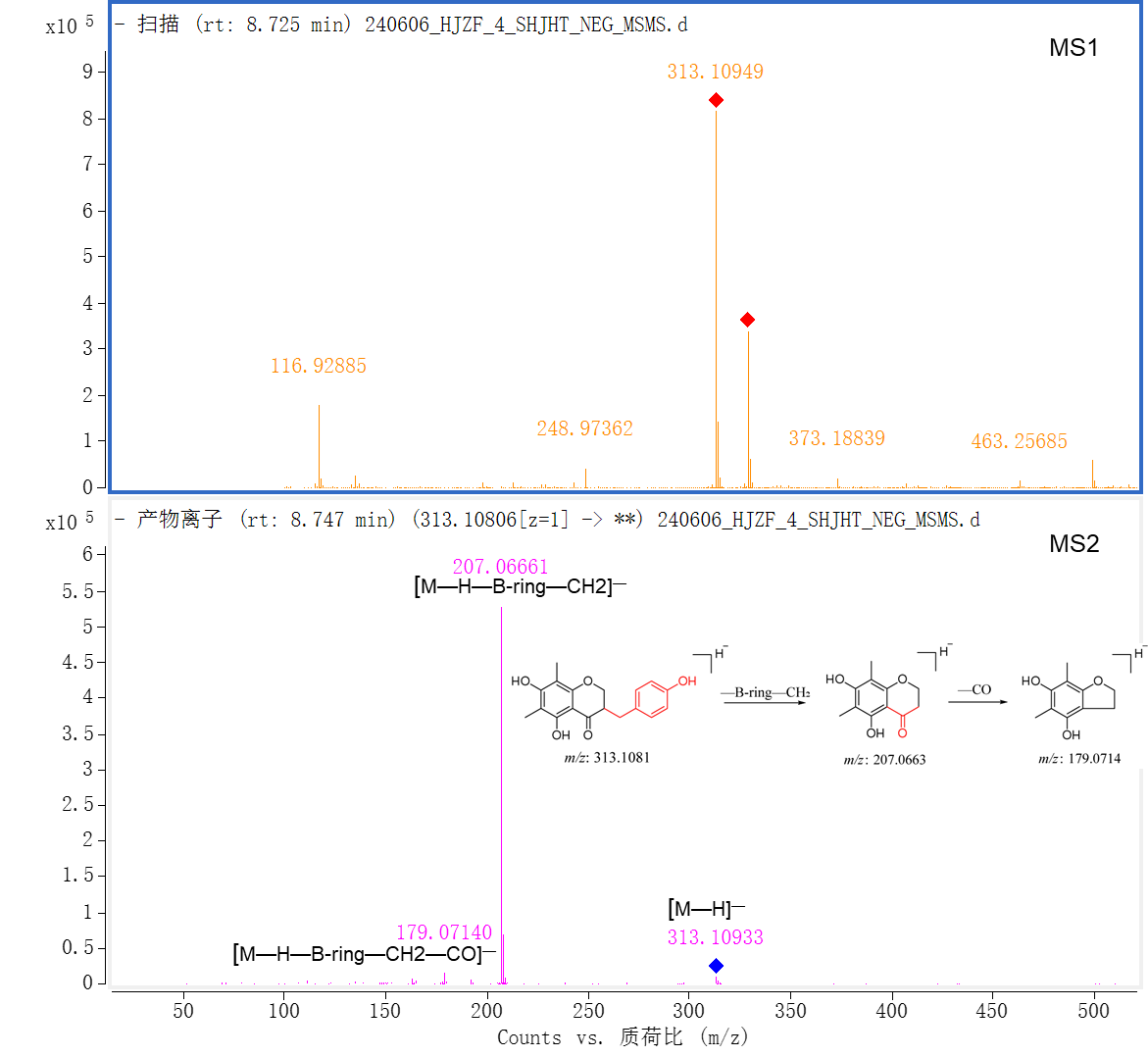
**

**Figure S3**. Mass spectrometry for detection and analysis of HIF.


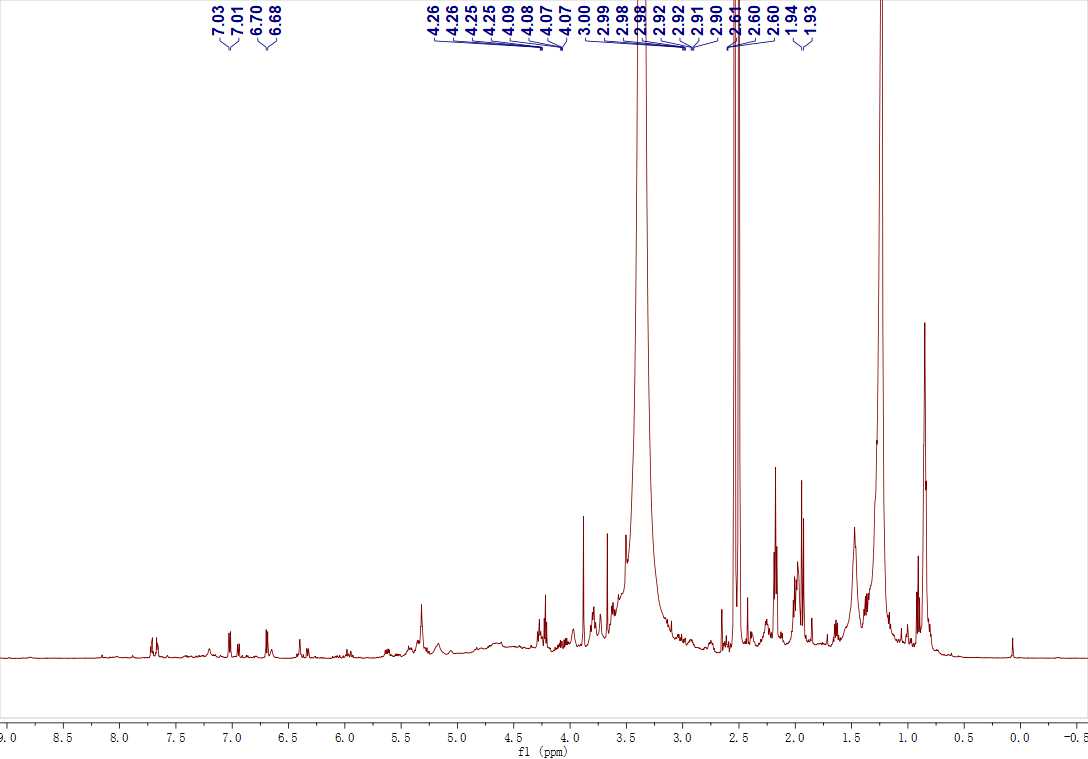


**Figure S4**. ^1^H NMR spectrum of HIF.


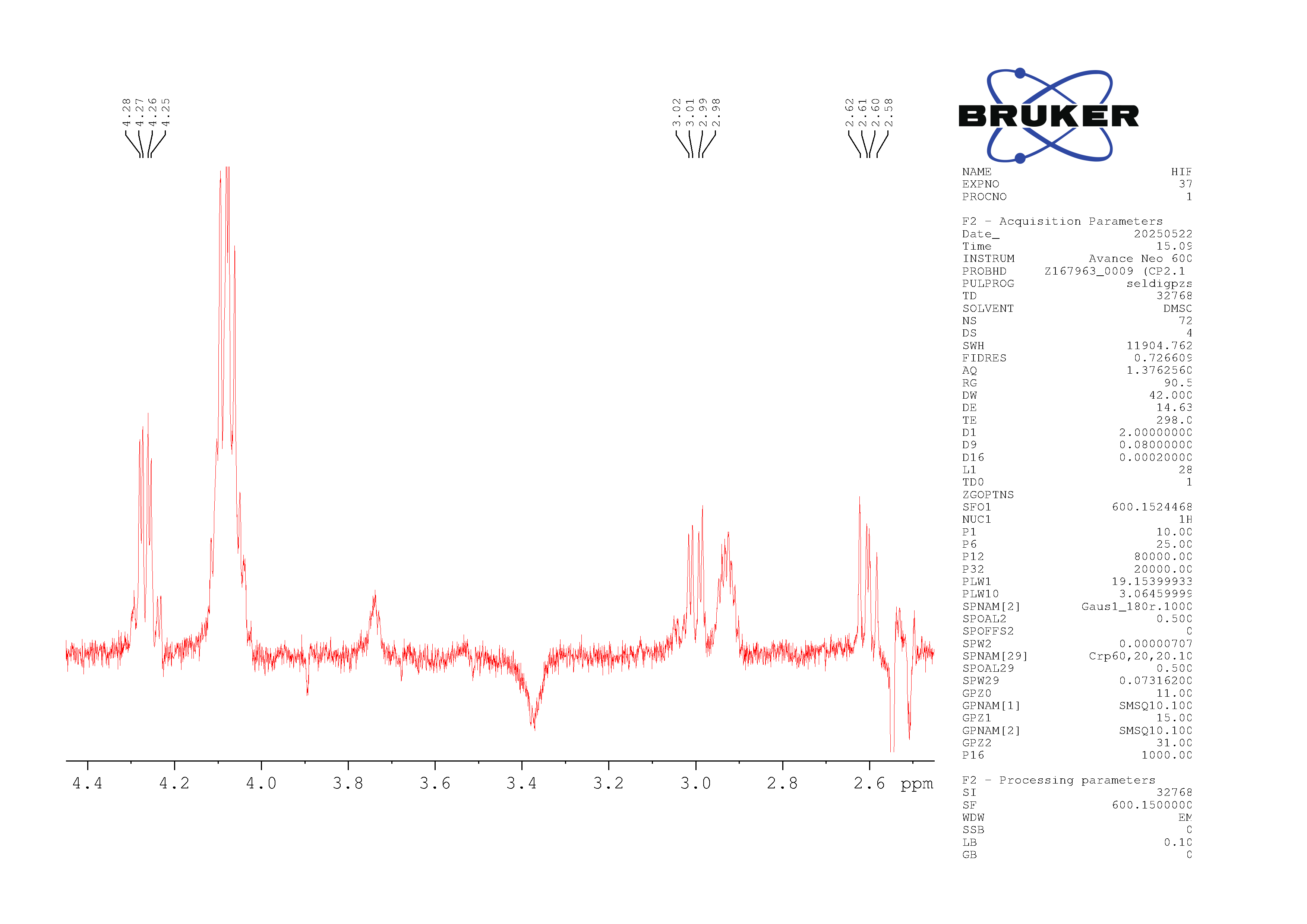


**Figure S5**. The selective one-dimensional (1D) ^1^H-^1^H TOCSY NMR spectrum, obtained by selective excitation at 4.067 ppm, revealed characteristic multiplet signals of the target structure at 4.25 ppm (doublet of doublets, dd), 2.99 ppm (dd), 2.92 ppm (multiplet, m) and 2.6 ppm (dd).


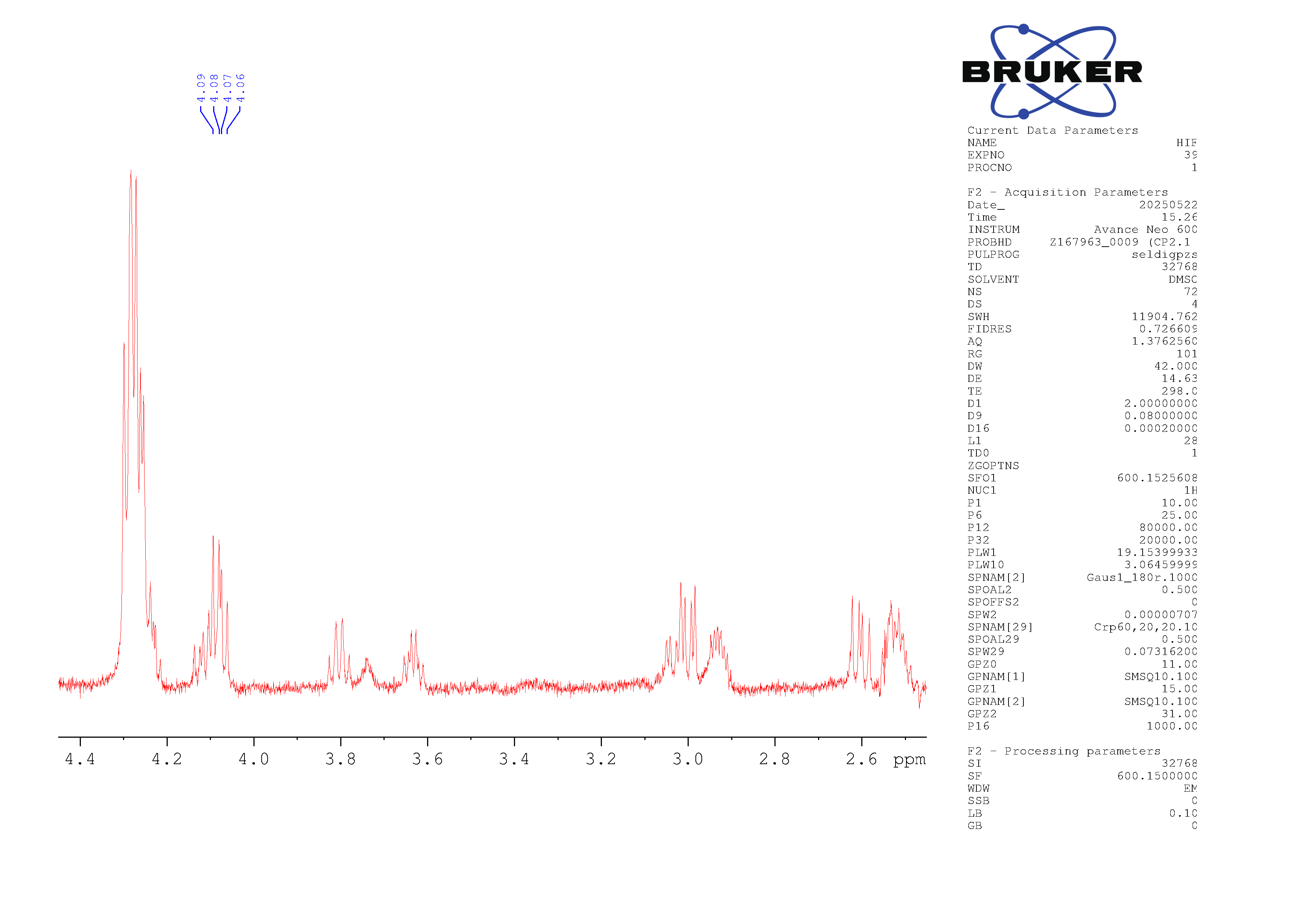


**Figure** **S6**. The selective 1D ^1^H-^1^H TOCSY NMR spectrum, acquired with selective excitation at 4.277 ppm, revealed a characteristic doublet of doublet (dd) at 4.07 ppm.


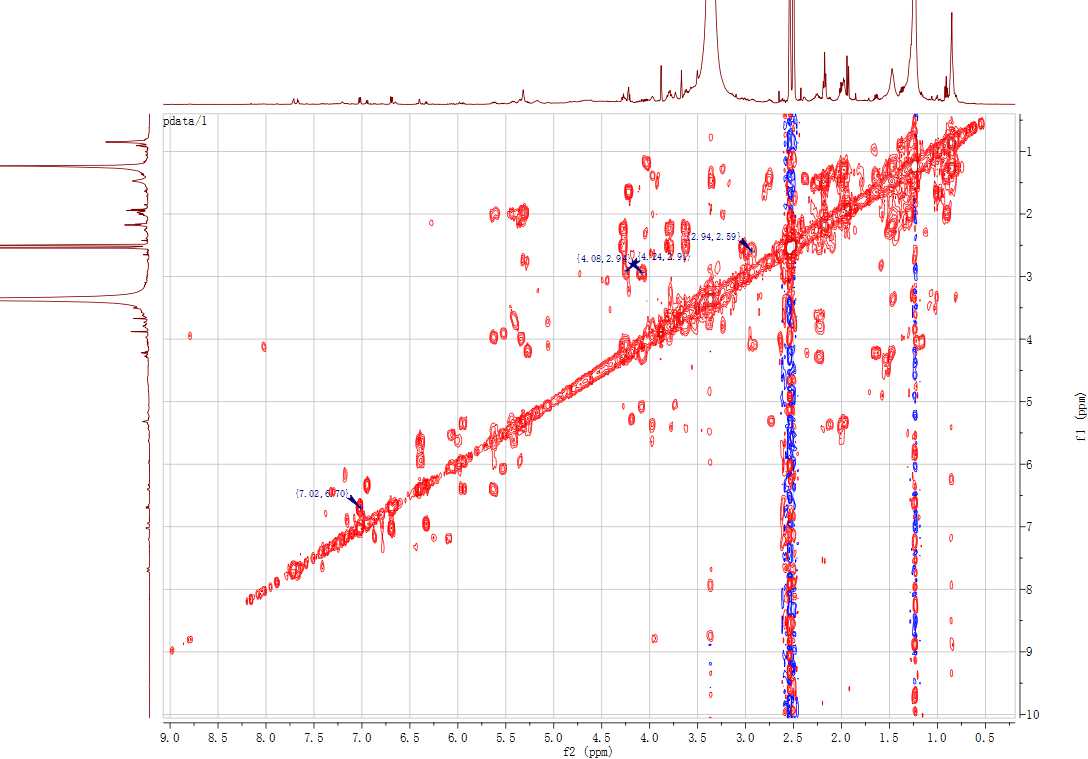


**Figure** **S7**. The two-dimensional ^1^H-^1^H COSY spectrum of the target compound, with cross-peaks corresponding to the relevant spin systems labeled for structural elucidation.


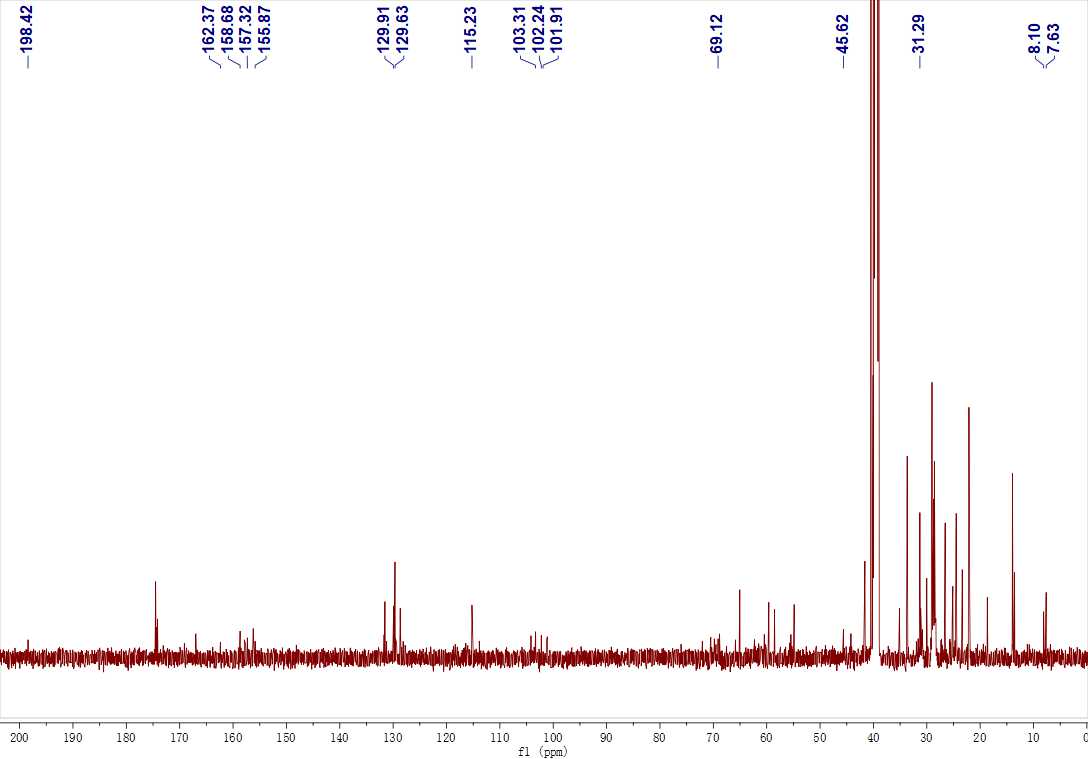


**Figure** **S8**. ^13^C NMR spectrum of HIF, with chemical shifts of relevant carbon atoms labeled for structural elucidation.


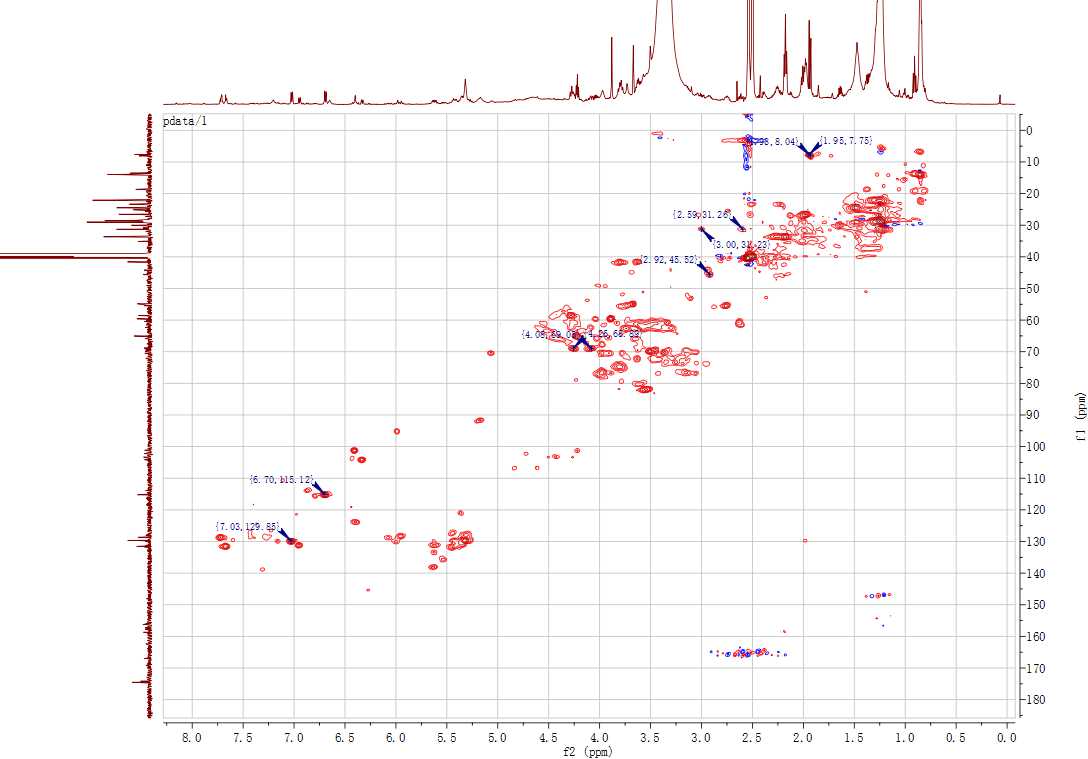


**Figure** **S9**. ^1^H-^13^C HSQC spectrum of HIF, with relevant cross-peaks labeled to aid in the assignment of proton-carbon correlations.


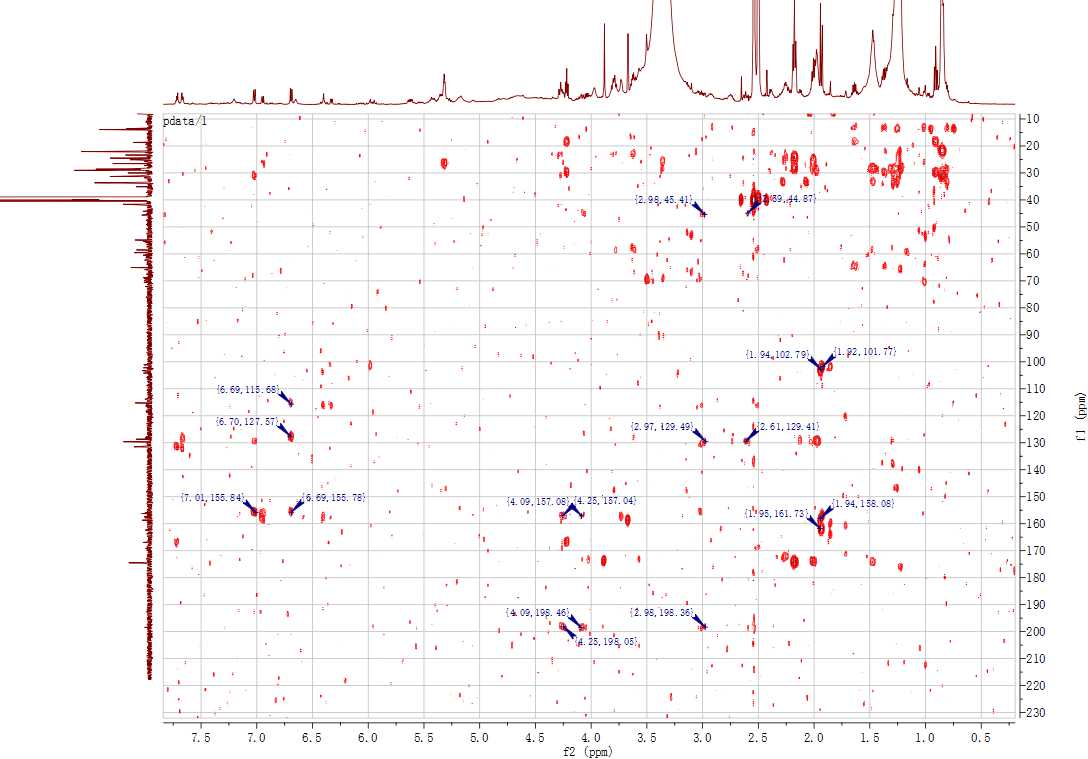


**Figure** **S10**. ^1^H-^13^C HMBC spectrum of HIF, with key cross-peaks labeled. Notably, the correlations observed at 4.09/157.08 ppm, 4.25/157.04 ppm and 1.94/158.08 ppm indicate long-range heteronuclear couplings between the methyl protons at positions 8 and 6 (8**-**CH_3_ or 6**-**CH_3_) and the methylene carbon at position 2 (2**-**CH_2_**-**).

**Figure** **S11**. Assigned chemical structure of the homoisoflavonoid compound (HIF), as determined by comprehensive spectroscopic analyses including LC-MS, NMR, and 2D correlation experiments.


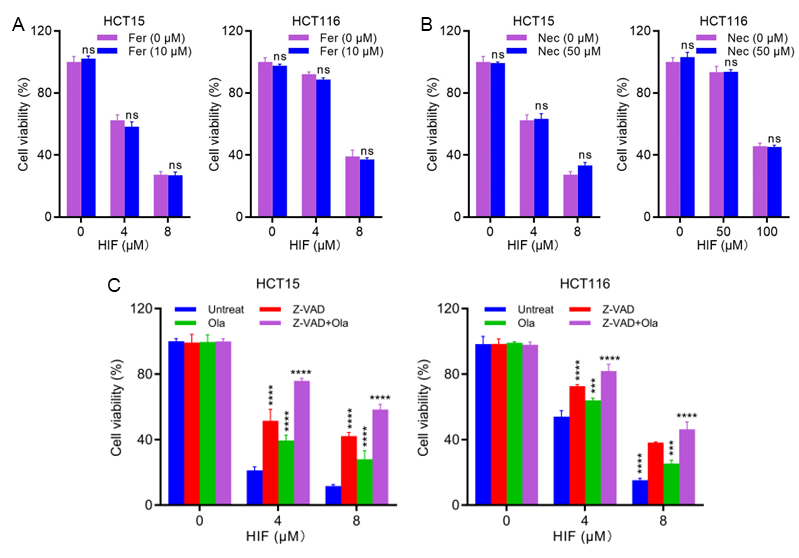


**Figure** **S12**. Effect of HIF and cell death pathway inhibitors on viability of HCT15 and HCT116 cells. A-C) Cell viability of HCT15 and HCT116 cells treated with HIF alone or in combination with various inhibitors, including Ferrostatin-1 (Fer), Necrostatin-1 (Nec), Z-VAD-FMK (Z-VAD), Olaparib (Ola), or the combined treatment of Z-VAD and Ola. *** P < 0.001, **** P < 0.0001; ns, not significant.


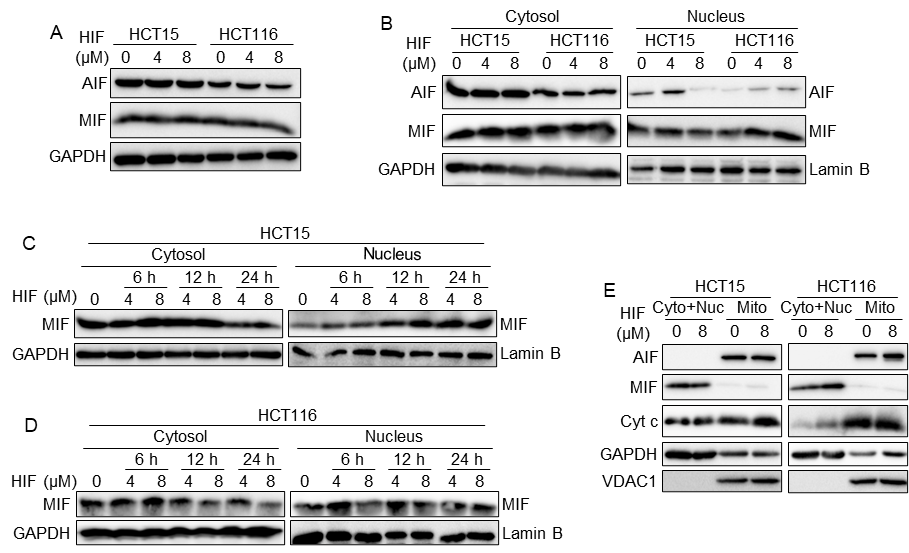


**Figure** **S13**. Protein expression levels and subcellular localization of AIF, MIF, and Cyt c following HIF treatment in colorectal cancer cells. A) Protein expression levels of AIF and MIF in HCT15 and HCT116 cells following 24 h of HIF treatment. B) Subcellular localization of AIF and MIF in cytosolic and nuclear fractions after 24 h of HIF treatment. C, D) Time-course analysis of MIF protein levels in the cytosolic and nuclear fractions of HCT15 and HCT116 cells at 6 h, 12 h and 24 h post-HIF treatment. E) Distribution of AIF, MIF and Cyt c in mitochondrial versus non-mitochondrial (cytosolic and nuclear) fractions following 24 h of HIF treatment in HCT15 and HCT116 cells.


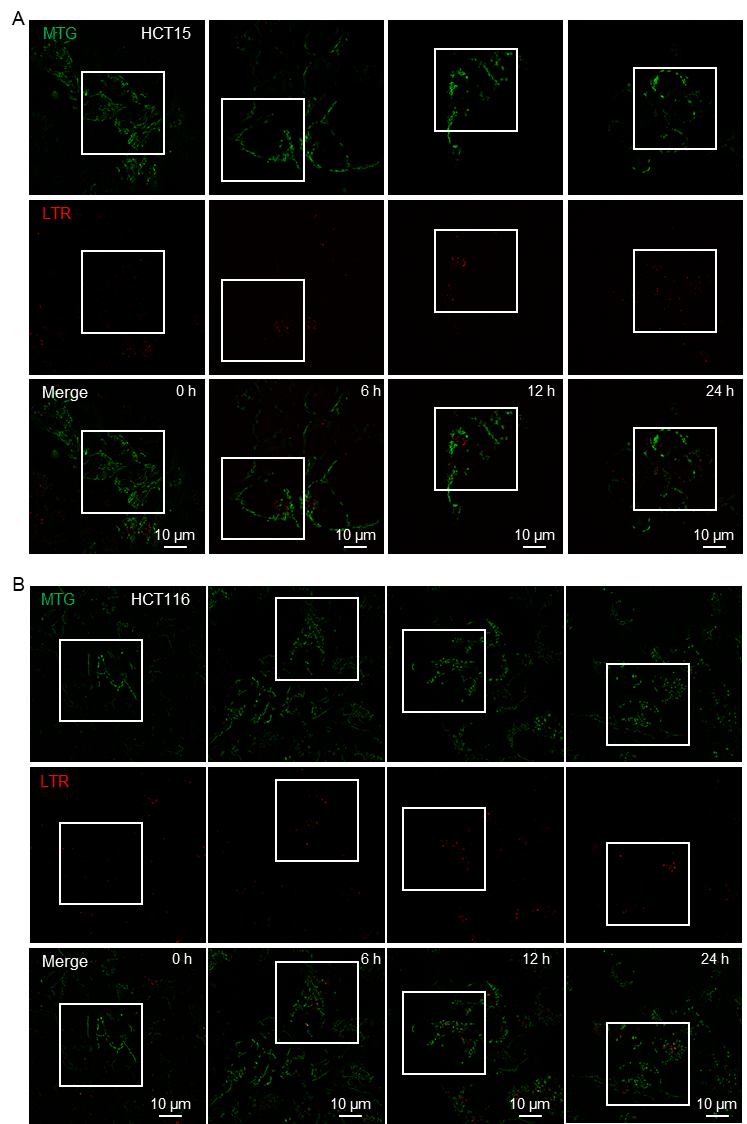


**Figure** **S14**. Structured illumination microscopy (SIM) images of HCT15 and HCT116 cells stained with MitoTracker and LysoTracker following HIF treatment at 0 h, 6 h, 12 h and 24 h. The area highlighted by the white square indicates the region magnified in Figure 7A and E.


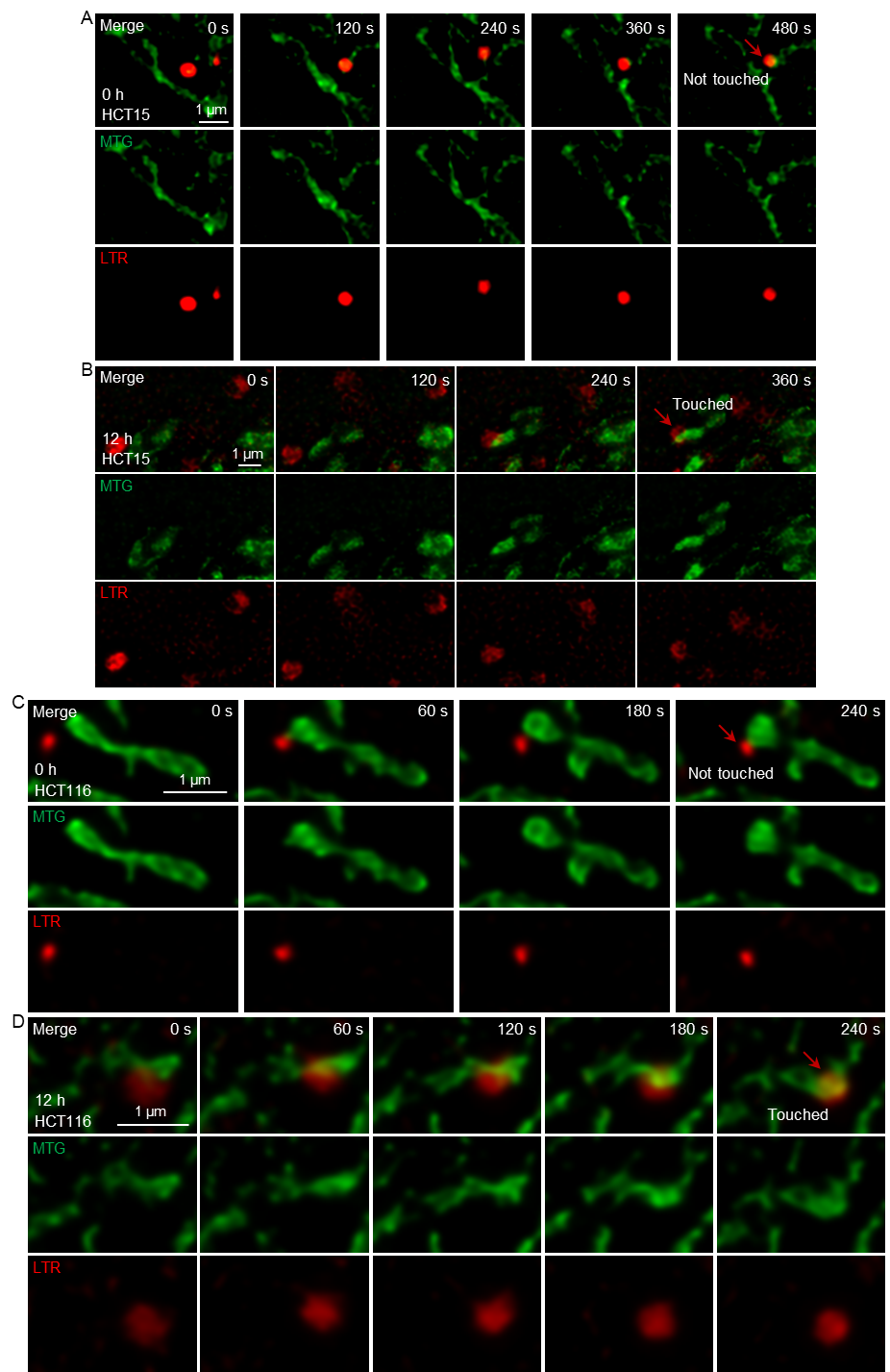


**Figure** **S15**. Time-lapse imaging of mitochondrial-lysosomal interactions in HCT15 and HCT116 cells after HIF treatment for 24 h.

**Table S1.** Summary of relevant NMR experimental parameters and their values.

| Spectra | Pulse sequence | D1 | Aq. | Expt. | TD |
| --- | --- | --- | --- | --- | --- |
| ^1^H | zg30 | 1s | 2.7525s | 2m8s | 65536 |
| ^13^C | Zgpg30 | 2s | 0.9175s | 51m4s | 65536 |
| ^1^H-^1^H COSY | cosygpppqf | 2s | F2:0.13s  F1:16.38ms | 5m13s | 2048*128 |
| ^1^H-^13^C HSQC | hsqcetgpsi2 | 1.5s | F2:0.13s  F1:16.38ms | 54m6s | 1024*256 |
| ^1^H-^13^C HMBC | hmbcgplpndqf | 1.5s | F2:0.45s  F1:3.39ms | 2h34m54s | 8192*128 |
| Selective 1D-TOCSY | seldigpzs | 2s | 1.376s | 4m34s | 32768 |

**Note:** The abbreviations “h”, “m”, and “s” represent hour, minute, and second, respectively. “D1” denotes the relaxation delay, “Aq” the acquisition time, “Expt” the total experimental duration, and “TD” the number of data points in the free induction decay (FID).

**Table S2.** Assignments of HIF were made based on multidimensional NMR data, with relevant correlations confirmed via ¹H**-**¹H COSY, ¹H**-**¹³C HSQC, and ¹H**-**¹³C HMBC spectra.

| **No.** | *δ*_H_ | *δ***_C_** |
| --- | --- | --- |
| 2 | 4.07, dd (8.6, 11.3)  4.25, dd (4.1, 11.4) | 69.1 |
| 3 | 2.92, m | 45.6 |
| 4 |  | 198.4 |
| 4a |  | 102.2 |
| 5 |  | 158.7 |
| 6 |  | 103.3 |
| 7 |  | 162.4 |
| 8 |  | 101.9 |
| 8a |  | 157.3 |
| 9 | 2.60, dd (13.6，9.8)  2.99, dd (14.1，5.1) | 31.3 |
| 1’ |  | 129.6 |
| 2’ | 7.02, d (8.4) | 129.9 |
| 3’ | 6.69, d (8.4) | 115.2 |
| 4’ |  | 155.9 |
| 5’ | 6.69, d (8.4) | 115.2 |
| 6’ | 7.02, d (8.4) | 129.9 |
| 6-CH_3_ | 1.94, s | 7.6 |
| 8-CH_3_ | 1.93, s | 8.1 |
